# A 16-Year Cohort Analysis of Autism Spectrum Disorder-Associated Morbidity in a Pediatric Population

**DOI:** 10.3389/fpsyt.2018.00635

**Published:** 2018-11-29

**Authors:** David Cawthorpe

**Affiliations:** Cumming School of Medicine, Departments of Psychiatry and Community Health Sciences, Institute for Child and Maternal Health, The University of Calgary, Calgary, AB, Canada

**Keywords:** autism spectrum disorder, temporal hypermorbidity, population cohort, ICD-9, ASD-associated morbidity

## Abstract

**Introduction:** This chapter presents the analysis of physician-diagnosed International Classification of Diseases (ICD version 9) disorders and diseases associated with autism spectrum disorders (ASD) in a 16-year pediatric cohort.

**Materials and Methods:** The sample (*n* = 47,180; 62% male) consisted of children in the Alberta Health Services Calgary Health Region catchment under the age of 3 years, who received any physician-assigned ICD 9 diagnosis before the age of three between April 1993 and December 31, 1994. There were 111 females and 609 males with ASD diagnosed at any time between 1993 and 2010. The results detail the 16-year odds ratio (OR) associations of ASD diagnosis within the major classes of international classification of diseases (ICD 9) stratified by age and sex in the cohort. Further, for those suffering from ASD and any other disorder or disease, the analysis presents by sex, age, and duration, the proportions of all index physician-assigned ICD diagnoses, arising significantly before and after the index ASD diagnosis.

**Results:** The rate of treated ASD in the cohort was 1 in 65 and the 16-year population rate of ASD was 62 per 10,000. For males with an ASD over the 16 year period, the ORs were significantly greater than the value one for 15 of the 17 main ICD classes and for 10 of the main ICD classes for females. Different age strata presented a more specific account of the main ICD class OR profiles. More specifically, 28 ICD disorders significantly preceded and 95 ICD disorders significantly followed ASD for females. Thirty-eight ICD disorders significantly preceded and 234 ICD disorders significantly followed ASD for males.

**Conclusions:** The results largely confirm past studies focusing on more constrained sets of ASD morbidity. The age-stratified ORs gauge the order of risk in time for the cohort. The proportions of specific ICD disorders arising before and after ASD may be useful in respect to informing basic ASD research and ASD clinical management. Limitations are discussed.

## Introduction

Between one in 65 and one in 88 children born in the United States and Canada are diagnosed with ASD (1–4). To date little detailed knowledge has emerged about the different types of morbidity associated with ASD. Based on a literature review spanning the period between 1991 and 2018, it was apparent that most of the ASD morbidity studies have generally focused only on concurrent or prodromal disorders, primarily psychiatric disorders, occasionally physical disorders, and at best only a few disorders within any given study (5–22). The literature review focused on titles containing the terms autism and the stem “morbid.” The literature illustrated a basic limitation of the morbidity research design to inform an understanding of the etiology of ASD or its clinical management.

### Background

While past studies have identified the ambiguity of comorbidity and multimorbidity definitions (23). Others have sought to remedy the situation, by expanding the conceptualization of comorbidity, multimorbidity, concurrent, and associated disorders. Progress has been made in terms of position papers identifying the scope of the definitions required to begin to understand the associations of disorders, both phenomenological and temporal (24, 25). Jakovljević, in a seminal paper, introduces the terms “anosognosia^1^” in relation to the medical field's approach to the study of multimorbidity, given its range and complexity and the observation that comorbidity and multimorbidity are “under-recognized, under-diagnosed, under-estimated and under-treated” (25). For example, a range of definitions for morbidity including comorbidity and multimorbidity have emerged to better communicate the concepts underpinning the potential relationships of disorders and diseases as these arise in the individual.

In its simplest form, comorbidity exists in from 35 to 88% of all ill people and refers to two or more concurrently co-existing diseases or disorders (25). Researchers have extended the possible definitions of morbidity, comorbidity, and multimorbidity to include terms such as hypercomorbidity and hypocomorbidity that refer to co-occurrence that is greater or lesser than chance, respectively (25). Time necessarily becomes a principle operator in distinguishing sets of definitions related to the directionality of comorbid diseases and their complex nature (26). Also of particular importance is that of each disease's probability in relation to the others' onset (26).

Multimorbidity is defined as the coexistence of multiple chronic diseases or conditions within an individual (27). The definitions of morbidity with inclusion of the often transient nature of temporal morbidity further complicates how morbidity is conceptualized. For example, disorders that are not always present or appear resolved in the individual either before or after the time of ASD onset, may be over-represented in the population of ASD-diagnosed individuals. Definition of morbidity, for the purpose of this paper, required extension to include the concept of temporality and associated transient diseases or disorders, not necessarily present at the time of index ASD diagnosis, yet present at some past or future time in proportions greater than that expected by chance alone. The present study extends previous work on ASD (28) in describing the full range of unique International Classification of Diseases (ICD) diagnoses associated in time with ASD.

Accompanying updated definitions aiding the conceptualization of morbidity is the advantage of access to large databases, such as the one on which present study and similar studies have been based (29–31). The 16-year database has been the source of numerous publications related to identifying key relationships between biomedical, physical, and mental disorders, in addition to the influence of the temporal occurrence of particular classes of disorders (e.g., mental disorders) in relationship to serious biomedical and somatic disorders (28–33). Identifying a cohort under the age of 3 years within a time frame and following the progress of disorders diagnosed within this group over a 16-year period made possible the examination of the relative emergence of temporal morbidity in relation to the index diagnosis of ASD.

Many disorders are transient (e.g., infections), however, once diagnosed, ASD persists as a comorbid diagnosis in time with all subsequent diagnoses.

### Study objective

The proposition under study is that many temporal physical and biomedical morbidities, transient or persistent, will arise in significant proportions within the ASD-diagnosed population in comparison to those without ASD. When over-represented (hypermorbidity), the pattern of morbidity associated with specific diagnoses in addition to main classes, may provide a better understanding of the etiology or the sequelae of ASD. Etiology might inform basic research *via* the study of specific infections and /or inflammatory processes as well as early diagnosis. Sequelae may facilitate ASD management and care planning when diseases or disorder appear above a given threshold. Of note is that by definition, representation of associated disease might arise in at least one of four main categories in relation to a target (pivot) diagnosis, ASD in this case: significant and non-representative of the sample, non-significant, or significantly before or after an index “pivot” diagnosis (e.g., ASD). Additionally, yet beyond the scope of the present study are the additional categories of transient *vs*. persistent.

## Materials and methods

The sample from which the sub-sample for this study^2^ derives and the methods employed in this study have been described in detail (28–31, 34). This study extends previous work in describing the specific disorders arising significantly before or after index ASD, the pivot diagnosis.

For this study, a sample was constructed consisting of a regional cohort under 3 years of age between April 1, 1993 and December 31, 1994 having presented with any physician-diagnosis. All ICD 9 diagnoses assigned to this set of unique individuals over the next approximately 16 years up to November 2010 were merged by unique individual and truncated at maximum age <18 years. Those having an ASD diagnosis were labeled as a group along with all their associated ICD diagnoses, and individuals with unlinked diagnoses making up the comparison group.

The odds ratios (OR) were calculated for this group in relation to the remaining 17 main classes of ICD diseases and disorders (excluding mental disorders). The standard OR formula takes the form of ad/bc, with each of the cells representing a (neither condition), b (one condition), c (the other condition), and d (both conditions) in the 2X2 cross-tabulation. The results were represented in tables and graphs separately for each sex and stratified by age groups. ORs with lower or upper 95% confidence intervals (LCI, UCI) greater or less than the value one were considered to be statistically significant (*p* < 0.05) with z set at value 1.96. Significance and 95% confidence intervals are noted in each table but not in the graphics. In the graphics, lines for each age strata connect the different main ICD classes for ease of comparison within and between age strata.

For those diagnosed with both ASD and any ICD disorder or disease, a count of the occurrence of each ICD diagnosis arising before and after the pivot ASD diagnosis were calculated within each diagnosis and the proportions arising before and after ASD were compared based on upper and lower 95% confidence intervals defined by the standard formula. Additionally, the average duration in days arising before and after ASD, and the average age in years of each disorder were calculated and tabled. Due to the large number of ICD diagnoses (>900), only those with significant proportions arising before and after ASD were tabled. Where the value zero occurred in a cell, the value one was substituted for the purpose of estimating either ORs or proportions and their 95% CIs. In determining the importance of a particular association both the proportion before or after ASD and the proportion before or after of the total sample need to be taken into account.

Note that in the tables the all-age group OR and cell values were the same as the < 3 age group OR cell values due to the method used to construct the sample. For example, the only individuals that could be linked in subsequent ages >3 years of age were the individuals within the cohort that remained in the catchment and received a physician-assigned diagnosis at any time over the next 16 years. Variations in the other age groups' ORs and cell values represent actual variations for these main classes of ICD disorders within the age groupings of the cohort over time. While attrition is in this sense may be confounded with absence of disease, the presence of a significant and representative OR association is actual.

Mental disorders were not considered in the present analysis in terms of the calculated ORs, as the different algorithm structure required for analysis was beyond the scope and resources of the present study. Including the temporal morbidity of specific mental disorders other than ASD was possible as output due to the logic structure in the algorithm giving rise to the examination of the specific proportions of ICD diagnoses arising before or after ASD (Table 4 in Supplementary Material).

## Results

There were 17,898 females in the 1993–1994 cohort sample with an average of 61 physician-diagnoses and a range between 1 and 754 diagnoses over the next 16 years for a total of 1,092,752 diagnoses. There were 111 females with ASD diagnosed at some point over the next 16 years. The female mean age of first ASD diagnosis was 7.5 years (std. dev. 3.6) with range from 0 to 16 years.

There were 29,191 males in the sample with an average of 66 physician-diagnoses and a range between 1 and 1,548 diagnoses for a 16-year total of 1,914,955 diagnoses. There were 609 males with ASD diagnosed at some point over the next 16 years. The male mean age of first ASD diagnosis was 7.8 years (std. dev. 3.2) with range from 0 to 17 years.

Of the total 16-year sample (all ages) one in 65 children in the 1993–94 cohort had an ASD diagnosis. The overall all-age OR comparison for the presence or absence of ASD by major classes of ICD diagnoses for males and females (ranked from highest to lowest by males) are shown in Table 1. For males with an ASD over the 16 year period, the ORs were significantly greater than one for all ICD main classes, with the exception of injury poisoning and complications of pregnancy and childbirth. The ORs for females with an ASD over the 16 year period were significantly greater than the value one for ICD main classes: sense organs, nervous system, circulatory system, congenital anomalies, endocrine, digestive system, skin subcutaneous tissue, neoplasms, genitourinary system, and injury/poisoning. The ORs for males with ASD were substantially higher than females for the ICD major classes: sense organs, respiratory system, and symptoms signs ill-defined conditions. The ORs for females with ASD were slightly higher than males for the ICD major classes: nervous system, circulatory system, congenital anomalies, endocrine, digestive system, skin subcutaneous tissue, neoplasms, genitourinary system, and injury/poisoning,

**Table 1:** The all-age odds ratios comparison for the presence and absence of ASD by major classes of ICD 9 diagnoses ranked from highest to lowest by males.

**Odds ratio**	**Males (*n* = 29,181)**	**Females (*n* = 17,898)**
Sense organs	4.94^*^	2.36^*^
Respiratory system	3.68^*^	2.21^ns^
Symptoms signs ill-defined conditions	3.33^*^	1.32^ns^
Nervous system	2.44^*^	3.22^*^
Circulatory system	1.7^*^	2.2^*^
Congenital anomalies	1.69^*^	2.58^*^
Endocrine	1.58^*^	2.15^*^
Digestive system	1.56^*^	1.92^*^
Perinatal conditions	1.54^*^	1.31^ns^
Musculoskeletal system connective tissue	1.4^*^	1.25^ns^
Blood-blood-forming organs	1.36^*^	1.12^ns^
Skin subcutaneous tissue	1.34^*^	1.7^*^
Neoplasms	1.29^*^	1.66^*^
Genitourinary system	1.29^*^	1.71^*^
Infectious-parasitic diseases	1.28^*^	1.44^ns^
Injury poisoning	1.22^ns^	1.86^*^
Complications of pregnancy childbirth	0.86^ns^	1.56^ns^

* *p < 0.05, ns, not significant*.

**Figure 1:**
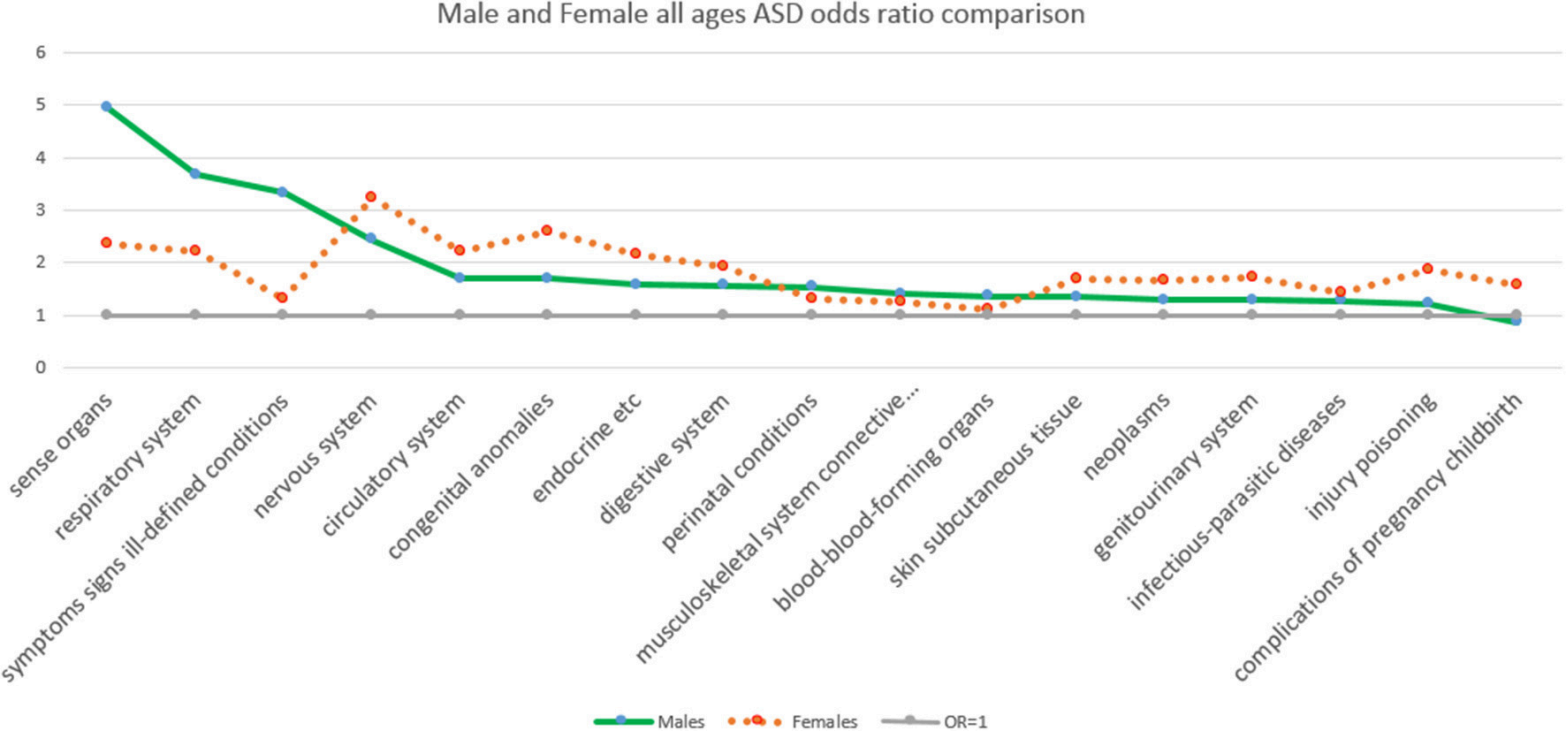
The odds ratios comparison of the presence and absence of ASD by major class ICD 9 diagnosis ranked by sex from highest to lowest by males.

Figure 1 shows for comparison the ASD OR values from Table 1 by major class ICD 9 diagnosis for males and females (all ages), ranked from highest to lowest by males. For the cohort across all ages, the greatest OR for males given an ASD over 16 year was sensory organs disorders, while for females it was nervous system disorders.

**Figure 2:**
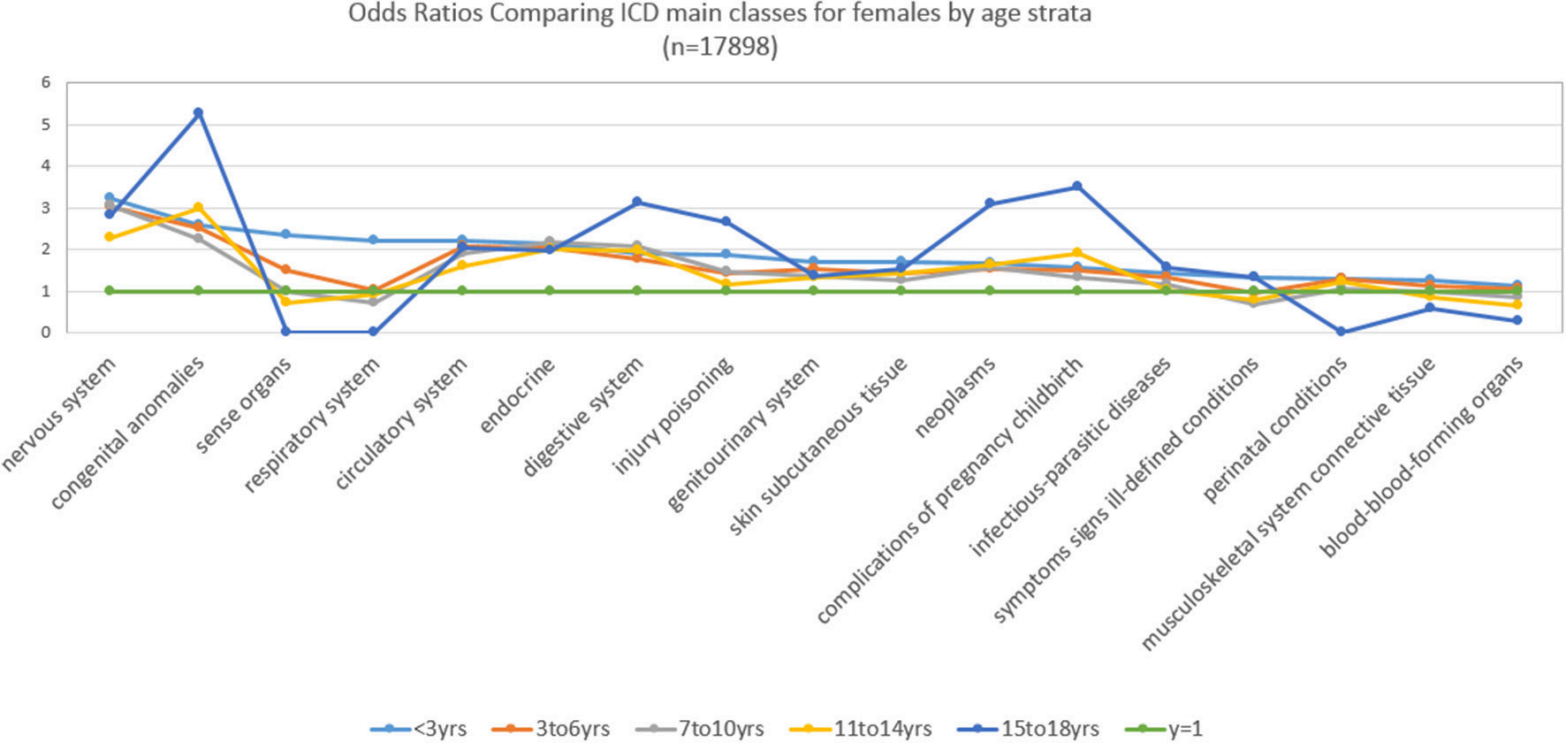
Main class ICD diagnoses odds ratios by age for females.

Figure 2 and Table 2 show for females with an ASD over 16 years, the ORs stratified by age groups, which present a somewhat different picture. For females, the ORs (value > 2) that were significantly and representatively greater at the earliest age for each main ICD class follows: 15–18 years, congenital anomalies; 15–18 years, complications of pregnancy childbirth; <3 years, nervous system; 15–18 years, neoplasms; 3–6 years, nervous system; <3 years, congenital anomalies; <3 years, sense organs; <3 years, circulatory system; <3 years, endocrine; 7–1 years, 0, digestive system.

**Table 2:** Main class ICD diagnoses odds ratios by mean age for females.

**Row**	**Mean age**	**ICD Main Class**	***a***	***b***	***c***	***d***	**Total**	**OR**	**LCI**	**UCI**	**OR>1**	**OR < 1**
1	0–18	Infectious–parasitic diseases	11,022	59	6,765	52	17,898	1.44	0.99	2.09	ns	ns
2	0–18	Neoplasms	15,703	91	2,084	20	17,898	1.66	1.02	2.69	^*^	ns
3	0–18	Endocrine	15,173	81	2,614	30	17,898	2.15	1.41	3.28	^*^	ns
4	0–18	Blood-blood-forming organs	16,488	102	1,299	9	17,898	1.12	0.57	2.22	ns	ns
5	0–18	Nervous system	16,382	87	1,405	24	17,898	3.22	2.04	5.07	^*^	ns
6	0–18	Sense organs	2,112	6	15,675	105	17,898	2.36	1.03	5.37	^*^	ns
7	0–18	Circulatory system	16,520	95	1,267	16	17,898	2.2	1.29	3.74	^*^	ns
8	0–18	Respiratory system	1,027	3	16,760	108	17,898	2.21	0.7	6.96	ns	ns
9	0–18	Digestive system	8,718	37	9,069	74	17,898	1.92	1.29	2.86	^*^	ns
10	0–18	Genitourinary system	10,535	51	7,252	60	17,898	1.71	1.18	2.49	^*^	ns
11	0–18	Complications of pregnancy childbirth	17,264	106	523	5	17,898	1.56	0.63	3.83	ns	ns
12	0–18	Skin subcutaneous tissue	4,175	17	13,612	94	17,898	1.7	1.01	2.85	^*^	ns
13	0–18	Musculoskeletal system connective tissue	10,285	58	7,502	53	17,898	1.25	0.86	1.82	ns	ns
14	0–18	Congenital anomalies	15,553	81	2,234	30	17,898	2.58	1.69	3.93	^*^	ns
15	0–18	Perinatal conditions	12,716	73	5,071	38	17,898	1.31	0.88	1.93	ns	ns
16	0–18	Symptoms signs ill-defined conditions	1,448	7	16,339	104	17,898	1.32	0.61	2.84	ns	ns
17	0–18	Injury poisoning	4,714	18	13,073	93	17,898	1.86	1.12	3.09	^*^	ns
18	<3	Infectious-parasitic diseases	11,022	59	6,765	52	17,898	1.44	0.99	2.09	ns	ns
19	<3	Neoplasms	15,703	91	2,084	20	17,898	1.66	1.02	2.69	^*^	ns
20	<3	Endocrine	15,173	81	2,614	30	17,898	2.15	1.41	3.28	^*^	ns
21	<3	Blood-blood-forming organs	16,488	102	1,299	9	17,898	1.12	0.57	2.22	ns	ns
22	<3	Nervous system	16,382	87	1,405	24	17,898	3.22	2.04	5.07	^*^	ns
23	<3	Sense organs	2,112	6	15,675	105	17,898	2.36	1.03	5.37	^*^	ns
24	<3	Circulatory system	16,520	95	1,267	16	17,898	2.2	1.29	3.74	^*^	ns
25	<3	Respiratory system	1,027	3	16,760	108	17,898	2.21	0.7	6.96	ns	ns
26	<3	Digestive system	8,718	37	9,069	74	17,898	1.92	1.29	2.86	^*^	ns
27	<3	Genitourinary system	10,535	51	7,252	60	17,898	1.71	1.18	2.49	^*^	ns
28	<3	Complications of pregnancy childbirth	17,264	106	523	5	17,898	1.56	0.63	3.83	ns	ns
29	<3	Skin subcutaneous tissue	4,175	17	13,612	94	17,898	1.7	1.01	2.85	^*^	ns
30	<3	Musculoskeletal system connective tissue	10,285	58	7,502	53	17,898	1.25	0.86	1.82	ns	ns
31	<3	Congenital anomalies	15,553	81	2,234	30	17,898	2.58	1.69	3.93	^*^	ns
32	<3	Perinatal conditions	12,716	73	5,071	38	17,898	1.31	0.88	1.93	ns	ns
33	<3	Symptoms signs ill-defined conditions	1,448	7	16,339	104	17,898	1.32	0.61	2.84	ns	ns
34	<3	Injury poisoning	4,714	18	13,073	93	17,898	1.86	1.12	3.09	^*^	ns
35	3–6	Infectious-parasitic diseases	9,707	58	6,435	52	16,252	1.35	0.93	1.97	ns	ns
36	3–6	Neoplasms	14,108	90	2,034	20	16,252	1.54	0.95	2.51	ns	ns
37	3–6	Endocrine	13,620	80	2,522	30	16,252	2.03	1.33	3.09	^*^	ns
38	3–6	Blood-blood-forming organs	14,877	101	1,265	9	16,252	1.05	0.53	2.08	ns	ns
39	3–6	Nervous system	14,784	86	1,358	24	16,252	3.04	1.93	4.79	^*^	ns
40	3–6	Sense organs	1,284	6	14,858	104	16,252	1.5	0.66	3.42	ns	ns
41	3–6	Circulatory system	14,920	94	1,222	16	16,252	2.08	1.22	3.54	^*^	ns
42	3–6	Respiratory system	458	3	15,684	107	16,252	1.04	0.33	3.29	ns	ns
43	3–6	Digestive system	7,455	36	8,687	74	16,252	1.76	1.18	2.63	^*^	ns
44	3–6	Genitourinary system	9,049	50	7,093	60	16,252	1.53	1.05	2.23	^*^	ns
45	3–6	Complications of pregnancy childbirth	15,650	105	492	5	16,252	1.51	0.61	3.73	ns	ns
46	3–6	Skin subcutaneous tissue	3,166	16	12,976	94	16,252	1.43	0.84	2.44	ns	ns
47	3–6	Musculoskeletal system connective tissue	8,820	57	7,322	53	16,252	1.12	0.77	1.63	ns	ns
48	3–6	Congenital anomalies	14,042	80	2,100	30	16,252	2.51	1.64	3.82	^*^	ns
49	3–6	Perinatal conditions	11,516	72	4,626	38	16,252	1.31	0.89	1.95	ns	ns
50	3–6	Symptoms signs ill-defined conditions	849	6	15,293	104	16,252	0.96	0.42	2.2	ns	ns
51	3–6	Injury poisoning	3,536	18	12,606	92	16,252	1.43	0.86	2.38	ns	ns
52	7–10	Infectious-parasitic diseases	7,490	50	5,568	44	13,152	1.18	0.79	1.78	ns	ns
53	7–10	Neoplasms	11,234	75	1,824	19	13,152	1.56	0.94	2.59	ns	ns
54	7–10	Endocrine	10,828	65	2,230	29	13,152	2.17	1.4	3.36	^*^	ns
55	7–10	Blood-blood-forming organs	11,924	87	1,134	7	13,152	0.85	0.39	1.83	ns	ns
56	7–10	Nervous system	11,806	71	1,252	23	13,152	3.05	1.9	4.91	^*^	ns
57	7–10	Sense organs	697	5	12,361	89	13,152	1	0.41	2.48	ns	ns
58	7–10	Circulatory system	11,956	80	1,102	14	13,152	1.9	1.07	3.36	^*^	ns
59	7–10	Respiratory system	205	2	12,853	92	13,152	0.73	0.18	3	ns	ns
60	7–10	Digestive system	5,607	25	7,451	69	13,152	2.08	1.31	3.29	^*^	ns
61	7–10	Genitourinary system	6,700	41	6,358	53	13,152	1.36	0.9	2.05	ns	ns
62	7–10	Complications of pregnancy childbirth	12,633	90	425	4	13,152	1.32	0.48	3.61	ns	ns
63	7–10	Skin subcutaneous tissue	2,022	12	11,036	82	13,152	1.25	0.68	2.3	ns	ns
64	7–10	Musculoskeletal system connective tissue	6,363	46	6,695	48	13,152	0.99	0.66	1.49	ns	ns
65	7–10	Congenital anomalies	11,323	70	1,735	24	13,152	2.24	1.4	3.57	^*^	ns
66	7–10	Perinatal conditions	9,362	66	3,696	28	13,152	1.07	0.69	1.67	ns	ns
67	7–10	Symptoms signs ill-defined conditions	481	5	12,577	89	13,152	0.68	0.28	1.68	ns	ns
68	7–10	Injury poisoning	2,127	11	10,931	83	13,152	1.47	0.78	2.76	ns	ns
69	11–14	Infectious-parasitic diseases	5,307	42	4,297	35	9,681	1.03	0.66	1.61	ns	ns
70	11–14	Neoplasms	8,098	59	1,506	18	9,681	1.64	0.96	2.79	ns	ns
71	11–14	Endocrine	7,843	53	1,761	24	9,681	2.02	1.24	3.28	^*^	ns
72	11–14	Blood-blood-forming organs	8,691	72	913	5	9,681	0.66	0.27	1.64	ns	ns
73	11–14	Nervous system	8,544	60	1,060	17	9,681	2.28	1.33	3.93	^*^	ns
74	11–14	Sense organs	364	4	9,240	73	9,681	0.72	0.26	1.98	ns	ns
75	11–14	Circulatory system	8,710	66	894	11	9,681	1.62	0.85	3.09	ns	ns
76	11–14	Respiratory system	116	1	9,488	76	9,681	0.93	0.13	6.74	ns	ns
77	11–14	Digestive system	3,954	20	5,650	57	9,681	1.99	1.2	3.32	^*^	ns
78	11–14	Genitourinary system	4,565	31	5,039	46	9,681	1.34	0.85	2.12	ns	ns
79	11–14	Complications of pregnancy childbirth	9,269	72	335	5	9,681	1.92	0.77	4.79	ns	ns
80	11–14	Skin subcutaneous tissue	1,201	7	8,403	70	9,681	1.43	0.66	3.12	ns	ns
81	11–14	Musculoskeletal system connective tissue	4,016	35	5,588	42	9,681	0.86	0.55	1.35	ns	ns
82	11–14	Congenital anomalies	8,349	53	1,255	24	9,681	3.01	1.85	4.9	^*^	ns
83	11–14	Perinatal conditions	7,011	53	2,593	24	9,681	1.22	0.75	1.99	ns	ns
84	11–14	Symptoms signs ill-defined conditions	294	3	9,310	74	9,681	0.78	0.24	2.49	ns	ns
85	11–14	Injury poisoning	1,153	8	8,451	69	9,681	1.18	0.56	2.45	ns	ns
86	15–18	Infectious-parasitic diseases	2,330	13	1,944	17	4,304	1.57	0.76	3.23	ns	ns
87	15–18	Neoplasms	3,515	18	759	12	4,304	3.09	1.48	6.44	^*^	ns
88	15–18	Endocrine	3,409	20	865	10	4,304	1.97	0.92	4.23	ns	ns
89	15–18	Blood-blood-forming organs	3,815	29	459	1	4,304	0.29	0.04	2.11	ns	ns
90	15–18	Nervous system	3,712	21	562	9	4,304	2.83	1.29	6.21	^*^	ns
91	15–18	Circulatory system	3,812	24	462	6	4,304	2.06	0.84	5.07	ns	ns
92	15–18	Digestive system	1,650	5	2,624	25	4,304	3.14	1.2	8.23	^*^	ns
93	15–18	Genitourinary system	1,728	10	2,546	20	4,304	1.36	0.63	2.91	ns	ns
94	15–18	Complications of pregnancy childbirth	4,095	26	179	4	4,304	3.52	1.22	10.19	^*^	ns
95	15–18	Skin subcutaneous tissue	426	2	3,848	28	4,304	1.55	0.37	6.53	ns	ns
96	15–18	Musculoskeletal system connective tissue	1,454	14	2,820	16	4,304	0.59	0.29	1.21	ns	ns
97	15–18	Congenital anomalies	3,733	17	541	13	4,304	5.28	2.55	10.92	^*^	ns
98	15–18	Perinatal conditions	3,358	22	916	8	4,304	1.33	0.59	3	ns	ns
99	15–18	Injury poisoning	356	1	3,918	29	4,304	2.64	0.36	19.4	ns	ns

* *p < 0.05, ns, not significant*.

**Figure 3:**
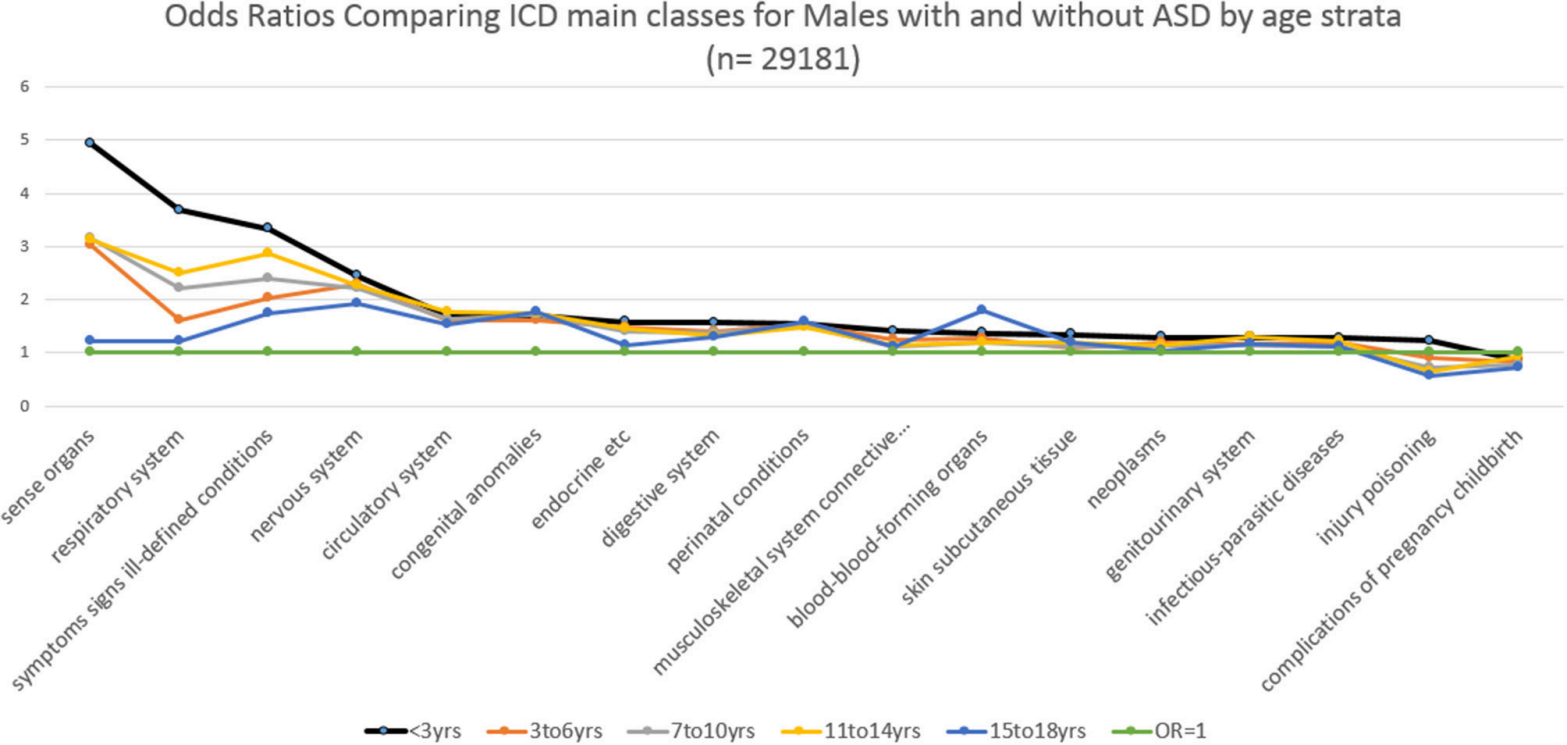
Main class ICD diagnoses odds ratios by age for males.

Similarly, Figure 3 and Table 3 show for males with an ASD over 16 years, the ORs stratified by age groups. For males, the ORs (value >2) that were significantly and representatively greater at the earliest age for each main ICD class follow: <3 years, sense organs; < 3 years, respiratory system; < 3 years, symptoms signs ill-defined conditions; 7–10 years, sense organs; 3–6 years, sense organs; < 3 years, nervous system; 7–10 years, symptoms signs ill-defined conditions; 3–6 years, nervous system. Additionally for males, one OR (value < 1) was significantly and representatively less at the earliest age 7–10 years. for injury and poisoning.

**Table 3:** Odds ratios by mean age for males.

**Row**	**Mean age**	**ICD Main Class**	***a***	***b***	***c***	***d***	**Total**	**OR**	**LCI**	**UCI**	**OR>1**	**OR < 1**
1	0–18	Infectious-parasitic diseases	19,886	387	8,702	216	29,191	1.28	1.08	1.51	^*^	ns
2	0–18	Neoplasms	25,411	519	3,177	84	29,191	1.29	1.03	1.63	^*^	ns
3	0–18	Endocrine	24,425	475	4,163	128	29,191	1.58	1.3	1.93	^*^	ns
4	0–18	Blood-blood-forming organs	26,219	537	2,369	66	29,191	1.36	1.05	1.76	^*^	ns
5	0–18	Nervous system	26,526	507	2,062	96	29,191	2.44	1.95	3.04	^*^	ns
6	0–18	Sense organs	3,005	14	25,583	589	29,191	4.94	2.9	8.41	^*^	ns
7	0–18	Circulatory system	26,448	530	2,140	73	29,191	1.7	1.33	2.18	^*^	ns
8	0–18	Respiratory system	1,508	9	27,080	594	29,191	3.68	1.9	7.11	^*^	ns
9	0–18	Digestive system	13,398	218	15,190	385	29,191	1.56	1.32	1.84	^*^	ns
10	0–18	Genitourinary system	18,286	349	10,302	254	29,191	1.29	1.1	1.52	^*^	ns
11	0–18	Complications of pregnancy childbirth	27,874	590	714	13	29,191	0.86	0.49	1.5	ns	ns
12	0–18	Skin subcutaneous tissue	6,874	115	21,714	488	29,191	1.34	1.09	1.65	^*^	ns
13	0–18	Musculoskeletal system connective tissue	16,670	301	11,918	302	29,191	1.4	1.19	1.65	^*^	ns
14	0–18	Congenital anomalies	24,002	456	4,586	147	29,191	1.69	1.4	2.04	^*^	ns
15	0–18	Perinatal conditions	19,411	349	9,177	254	29,191	1.54	1.31	1.81	^*^	ns
16	0–18	Symptoms signs ill-defined conditions	2,240	15	26,348	588	29,191	3.33	1.99	5.57	^*^	ns
17	0–18	Injury poisoning	6,051	109	22,537	494	29,191	1.22	0.99	1.5	ns	ns
18	<3	Infectious-parasitic diseases	19,886	387	8,702	216	29,191	1.28	1.08	1.51	^*^	ns
19	<3	Neoplasms	25,411	519	3,177	84	29,191	1.29	1.03	1.63	^*^	ns
20	<3	Endocrine	24,425	475	4,163	128	29,191	1.58	1.3	1.93	^*^	ns
21	<3	Blood-blood-forming organs	26,219	537	2,369	66	29,191	1.36	1.05	1.76	^*^	ns
22	<3	Nervous system	26,526	507	2,062	96	29,191	2.44	1.95	3.04	^*^	ns
23	<3	Sense organs	3,005	14	25,583	589	29,191	4.94	2.9	8.41	^*^	ns
24	<3	Circulatory system	26,448	530	2,140	73	29,191	1.7	1.33	2.18	^*^	ns
25	<3	Respiratory system	1,508	9	27,080	594	29,191	3.68	1.9	7.11	^*^	ns
26	<3	Digestive system	13,398	218	15,190	385	29,191	1.56	1.32	1.84	^*^	ns
27	<3	Genitourinary system	18,286	349	10,302	254	29,191	1.29	1.1	1.52	^*^	ns
28	<3	Complications of pregnancy childbirth	27,874	590	714	13	29,191	0.86	0.49	1.5	ns	ns
29	<3	Skin subcutaneous tissue	6,874	115	21,714	488	29,191	1.34	1.09	1.65	^*^	ns
30	<3	Musculoskeletal system connective tissue	16,670	301	11,918	302	29,191	1.4	1.19	1.65	^*^	ns
31	<3	Congenital anomalies	24,002	456	4,586	147	29,191	1.69	1.4	2.04	^*^	ns
32	<3	Perinatal conditions	19,411	349	9,177	254	29,191	1.54	1.31	1.81	^*^	ns
33	<3	Symptoms signs ill-defined conditions	2,240	15	26,348	588	29,191	3.33	1.99	5.57	^*^	ns
34	<3	Injury poisoning	6,051	109	22,537	494	29,191	1.22	0.99	1.5	ns	ns
35	3–6	Infectious-parasitic diseases	17,775	387	8,283	215	26,660	1.19	1.01	1.41	^*^	ns
36	3–6	Neoplasms	22,943	518	3,115	84	26,660	1.19	0.95	1.51	ns	ns
37	3–6	Endocrine	22,046	475	4,012	127	26,660	1.47	1.2	1.79	^*^	ns
38	3–6	Blood-blood-forming organs	23,750	536	2,308	66	26,660	1.27	0.98	1.64	ns	ns
39	3–6	Nervous system	24,065	506	1,993	96	26,660	2.29	1.83	2.86	^*^	ns
40	3–6	Sense organs	1,756	14	24,302	588	26,660	3.03	1.78	5.17	^*^	ns
41	3–6	Circulatory system	24,001	529	2,057	73	26,660	1.61	1.26	2.07	^*^	ns
42	3–6	Respiratory system	625	9	25,433	593	26,660	1.62	0.83	3.14	ns	ns
43	3–6	Digestive system	11,498	217	14,560	385	26,660	1.4	1.18	1.66	^*^	ns
44	3–6	Genitourinary system	16,078	349	9,980	253	26,660	1.17	0.99	1.38	ns	ns
45	3–6	Complications of pregnancy childbirth	25,377	589	681	13	26,660	0.82	0.47	1.43	ns	ns
46	3–6	Skin subcutaneous tissue	5,284	114	20,774	488	26,660	1.09	0.89	1.34	ns	ns
47	3–6	Musculoskeletal system connective tissue	14,396	300	11,662	302	26,660	1.24	1.06	1.46	^*^	ns
48	3–6	Congenital anomalies	21,736	455	4,322	147	26,660	1.62	1.35	1.96	^*^	ns
49	3–6	Perinatal conditions	17,661	349	8,397	253	26,660	1.52	1.29	1.8	^*^	ns
50	3–6	Symptoms signs ill–defined conditions	1,288	15	24,770	587	26,660	2.03	1.22	3.41	^*^	ns
51	3–6	Injury poisoning	4,277	108	21,781	494	26,660	0.9	0.73	1.11	ns	ns
52	7–10	Infectious-parasitic diseases	14,263	347	7,244	198	22,052	1.12	0.94	1.34	ns	ns
53	7–10	Neoplasms	18,679	464	2,828	81	22,052	1.15	0.91	1.46	ns	ns
54	7–10	Endocrine	17,908	425	3,599	120	22,052	1.4	1.14	1.73	^*^	ns
55	7–10	Blood-blood-forming organs	19,470	485	2,037	60	22,052	1.18	0.9	1.55	ns	ns
56	7–10	Nervous system	19,647	451	1,860	94	22,052	2.2	1.75	2.76	^*^	ns
57	7–10	Sense organs	1,078	9	20,429	536	22,052	3.14	1.62	6.09	^*^	ns
58	7–10	Circulatory system	19,648	472	1,859	73	22,052	1.63	1.27	2.1	^*^	ns
59	7–10	Respiratory system	345	4	21,162	541	22,052	2.2	0.82	5.93	ns	ns
60	7–10	Digestive system	8,848	185	12,659	360	22,052	1.36	1.14	1.63	^*^	ns
61	7–10	Genitourinary system	12,741	305	8,766	240	22,052	1.14	0.96	1.36	ns	ns
62	7–10	Complications of pregnancy childbirth	20,940	534	567	11	22,052	0.76	0.42	1.39	ns	ns
63	7–10	Skin subcutaneous tissue	3,675	86	17,832	459	22,052	1.1	0.87	1.39	ns	ns
64	7–10	Musculoskeletal system connective tissue	10,836	262	10,671	283	22,052	1.1	0.93	1.3	ns	ns
65	7–10	Congenital anomalies	17,900	406	3,607	139	22,052	1.7	1.4	2.07	^*^	ns
66	7–10	Perinatal conditions	14,580	318	6,927	227	22,052	1.5	1.26	1.79	^*^	ns
67	7–10	Symptoms signs ill–defined conditions	739	8	20,768	537	22,052	2.39	1.18	4.82	^*^	ns
68	7–10	Injury poisoning	2,584	87	18,923	458	22,052	0.72	0.57	0.91	ns	^*^
69	11–14	Infectious-parasitic diseases	9,858	235	5,509	160	15,762	1.22	0.99	1.49	ns	ns
70	11–14	Neoplasms	13,118	330	2,249	65	15,762	1.15	0.88	1.5	ns	ns
71	11–14	Endocrine	12,571	299	2,796	96	15,762	1.44	1.14	1.82	^*^	ns
72	11–14	Blood-blood-forming organs	13,784	347	1,583	48	15,762	1.2	0.89	1.64	ns	ns
73	11–14	Nervous system	13,834	316	1,533	79	15,762	2.26	1.75	2.9	^*^	ns
74	11–14	Sense organs	593	5	14,774	390	15,762	3.13	1.29	7.59	^*^	ns
75	11–14	Circulatory system	13,896	333	1,471	62	15,762	1.76	1.33	2.32	^*^	ns
76	11–14	Respiratory system	192	2	15,175	393	15,762	2.49	0.62	10.05	ns	ns
77	11–14	Digestive system	6,054	130	9,313	265	15,762	1.33	1.07	1.64	^*^	ns
78	11–14	Genitourinary system	8,773	200	6,594	195	15,762	1.3	1.06	1.58	^*^	ns
79	11–14	Complications of pregnancy childbirth	14,952	385	415	10	15,762	0.94	0.5	1.77	ns	ns
80	11–14	Skin subcutaneous tissue	2,221	49	13,146	346	15,762	1.19	0.88	1.61	ns	ns
81	11–14	Musculoskeletal system connective tissue	6,782	161	8,585	234	15,762	1.15	0.94	1.41	ns	ns
82	11–14	Congenital anomalies	12,869	295	2,498	100	15,762	1.75	1.39	2.2	^*^	ns
83	11–14	Perinatal conditions	10,667	239	4,700	156	15,762	1.48	1.21	1.82	^*^	ns
84	11–14	Symptoms signs ill-defined conditions	438	4	14,929	391	15,762	2.87	1.07	7.71	^*^	ns
85	11–14	Injury poisoning	1,245	47	14,122	348	15,762	0.65	0.48	0.89	ns	^*^
86	15–18	Infectious-parasitic diseases	3,610	93	2,093	60	5,856	1.11	0.8	1.55	ns	ns
87	15–18	Neoplasms	4,654	124	1,049	29	5,856	1.04	0.69	1.56	ns	ns
88	15–18	Endocrine	4,592	120	1,111	33	5,856	1.14	0.77	1.68	ns	ns
89	15–18	Blood-blood-forming organs	5,070	125	633	28	5,856	1.79	1.18	2.73	^*^	ns
90	15–18	Nervous system	4,990	120	713	33	5,856	1.92	1.3	2.85	^*^	ns
91	15–18	Sense organs	180	4	5,523	149	5,856	1.21	0.44	3.31	ns	ns
92	15–18	Circulatory system	5,083	129	620	24	5,856	1.53	0.98	2.38	ns	ns
93	15–18	Respiratory system	45	1	5,658	152	5,856	1.21	0.17	8.83	ns	ns
94	15–18	Digestive system	2,116	48	3,587	105	5,856	1.29	0.91	1.82	ns	ns
95	15–18	Genitourinary system	3,127	78	2,576	75	5,856	1.17	0.85	1.61	ns	ns
96	15–18	Complications of pregnancy childbirth	5,547	150	156	3	5,856	0.71	0.22	2.25	ns	ns
97	15–18	Skin subcutaneous tissue	604	14	5,099	139	5,856	1.18	0.67	2.05	ns	ns
98	15–18	Musculoskeletal system connective tissue	1,985	50	3,718	103	5,856	1.1	0.78	1.55	ns	ns
99	15–18	Congenital anomalies	4,747	113	956	40	5,856	1.76	1.22	2.54	^*^	ns
100	15–18	Perinatal conditions	4,334	102	1,369	51	5,856	1.58	1.12	2.23	^*^	ns
101	15–18	Symptoms signs ill–defined conditions	129	2	5,574	151	5,856	1.75	0.43	7.13	ns	ns
102	15–18	Injury poisoning	261	12	5,442	141	5,856	0.56	0.31	1.03	ns	ns

* *p < 0.05, ns, not significant*.

Table 4 in Supplementary Material is comprised of 4 sections showing in rows the comparisons of the proportions of distinct physician-assigned ICD diagnoses for males and females arising significantly before and after ASD. The average age in years of individuals within each row is shown together with the average duration in days the ASD arose before or after each ICD diagnosis. Table 4 in Supplementary Material is ordered by row number and the ascending numeric sequence of the ICD code for each disorder, first for females in rows 1–28 (28 disorders) where ASD rose significantly before the corresponding ICD disorder, second for females in rows 31–125 (95 disorders) where ASD rose significantly after the corresponding ICD disorder. For males in rows 126–165 (38 disorders), ASD rose significantly before the corresponding ICD disorder and finally, for males in rows 166–401 (234 disorders), where ASD rose significantly after the corresponding ICD disorder.

Examples of disorders with the highest sample proportions diagnosed after and before ASD follow. For females ASD diagnosis preceded the following disorders with the highest counts ranging from 44 to 7% of the females sample, respectively: psychoses of childhood (299), hyperkinetic syndrome (314), emotional disorder child/adol (313), neurotic disorders (300), conduct disturbance nec (312), depressive disorder nec (311), other viral disease (78), adjustment reaction (309), screen-heart/resp/gu disorder (V81), disorder of menstruation (626), gastritis and duodenitis (535).

For females ASD diagnosis came after the following disorders with the highest counts ranging from 74 to 30% of the females sample, respectively: health supervision child (V20), otitis media, suppur/nos (382), ac up resp inf multiple sites/nos (465), acute nasopharyngitis (460), general symptoms (780), single liveborn (V30), disorders of conjunctiva (372), specific develop delays (315), contact dermatitis (692), ill-defined intest inf (009), nonsuppur otitis media (381), ac bronchitis/bronchiol (466), nutrit/metab/devel symp (783), general medical exam (V70), candidiasis (112), and resp sys/other chest symp (786).

For males ASD diagnosis preceded the following disorders with the highest counts ranging from 47 to 7% of the male sample, respectively: psychoses of childhood (299), hyperkinetic syndrome (314), other viral disease (78), depressive disorder nec (311), other soft tissue disorder (729), screen-heart/resp/gu disorder (V81), and special symptom nec (307).

For males ASD diagnosis came after the following disorders with the highest counts ranging from 79 to 34% of the male sample, respectively: health supervision child (V20), otitis media, suppur/nos (382), ac up resp inf multiple sites/nos (465), acute nasopharyngitis (460), general symptoms (780), ill-defined intest inf (009), disorders of conjunctiva (372), acute bronchitis/bronchiol (466), specific develop delays (315), nonsuppur otitis media (381), acute pharyngitis (462), skin/other integument symp (782), asthma (493), resp sys/other chest symp (786), contact dermatitis (692).

## Summary of results

The results of the analysis of ASD and the main ICD classes of disease and disorder indicated a significant association that varied with age groups. Given the construction of the sample, the age groupings provided a proxy for the temporal progression of the observed class associations, for which some main class ORs were more pronounced at various ages for one or both sexes. The significant OR associations with main ICD classes were less frequent for females.

The analysis results for specific ICD diseases and disorders provided more precise information about the temporal relationship of each disorder and ASD. For example, concurrently examining the mean age, the number of cases, mean duration in days that the specific diagnosis arose either before or after ASD, together with the significance of their proportions provided additional information related to the temporality of etiology and sequelae of ASD morbidity, especially when the whole sample proportions (e.g., >30%) the before or after ASD counts of each diagnosis were additionally examined for males and females.

## Discussion

The estimated prevalence of ASD diagnosis in the cohort sample of treated children was one in 65, and as estimated from the previous study based on those under the age of 19 years (28) was 62 per 10,000 in the entire population, a result similar to other United States and Canadian studies (1, 2) and higher than past studies (1, 35). One difference was that prevalence in the present 1993-94 cohort was prospective in that the value was calculated based on an ASD diagnosis made at any time over the next 16 years. An observation about Canadian data is that there have been few datasets that permit a conclusive estimate of ASD prevalence (36). The average age of ASD diagnosis in the present study was older than that previously reported for all of Alberta (36). The different study purposes, foci, and designs may have accounted for observed differences in age of diagnosis. The provincial study focused on the prospective Alberta Perinatal Health Program cohort in which the births were actively monitored over the course of the study (36), while the present study focused on the 16-year main ICD class and specific ICD disorder temporal morbidity associated with ASD in a group under 3-years of age between 1993 and 1995.

The emerging field of ASD comorbidity and multimorbidity is evolving and complex. The changing concept of morbidity must necessarily shift health systems and education to configure around multimorbidity from a focus on individual diseases (37). Multimorbidity is the most common chronic condition experienced in significant proportions by older and younger adults: Health system redesign is required to accommodate the emerging body of research (27). Systematic review indicates that the ability to respond clinically to morbidity, comorbidity, and multimorbidity is in its infancy (38).

A number of studies have focused attention on physical disorder comorbidity (39–44) including population studies (45, 46), putative mechanisms underpinning ASD (47–57), and how cross-comorbidity study identifies disease linkage (58), as well as autism-related genes (59) with the potential to identify cause (60).

For basic researchers Table 4 in Supplementary Material might serve as a reference for comparison to other population studies and inform deeper analysis that might provide more insight into the ASD etiology or sequelae, or both. It is important to take into account when reviewing the proportions of the sample with ASD as the pivot diagnosis, the raw counts also represent a portion of the total female or male sample. For example, in Table 4 (Supplementary Material), row 31, ASD followed 74% of female cases (*n* = 43 of 51) with ill-defined intestinal infection (009) representing 39% (*n* = 43 of 111) of the female sample with that particular disorder. In Table 4 (Supplementary Material), row 169, for males ill-defined intestinal infection (009) arose in 85% (*n* = 335 of 393) representing 55% (335 of 609) of the sample. For both males and females, the average age was 7 years and the average duration between the diagnosis ill-defined intestinal infection (009) and ASD was 1,885 and 1,890 days, respectively. The closeness of the age and average duration before ASD for both males and females is interesting, given the preponderance of males that suffer from ASD. The brain-gut axis is an increasing popular focus of research in recent years (60–62). It is possibly mere coincidence, even though many infectious and allergic diseases in the sample had this same profile, that males and females in a population are diagnosed with ASD about the same period on average after suffering from this gastro-intestinal disorder. Similarly, the present study also identifies that sensory and respiratory disorders occur with substantial magnitude before ASD. In respect to development of biome *via* maternal inoculation *via* natural vaginal delivery, study has identified those born under conditions of augmented birth (e.g., cesarean section) are at higher risk of ASD (3). In alignment, the present study indicates that ASD children may have compromised immune systems, possibly associated with the brain-gut axis (61). Comparing the present study findings to other studies focusing on morbidity, there is similar overlap in the association of inflammatory processes, infection, and the emergence of psychiatric disorders thought to have organic origins, such as schizophrenia (63–65).

How might a clinical temporal, transient, hypercomorbidity profile case stack up, against Table 4 in Supplementary Material? The present work may contribute in the following ways. In clinical practice repeated visits of any child above a threshold (possibly the before or after counts of disorders in Table 4 (Supplementary Material)) may signal the need for more detailed investigation, such as thorough assessment of the child's having attained developmental milestones. This seems a sweeping and costly generalization for any practice guide based on a single diagnosis. However, by examining any given child's etiological morbidity profile across multiple diseases and disorders might possibly reveal a more valid constellation of thresholds, the limitations of the present study notwithstanding. In practical terms, the proportions presented in Table 4 (Supplementary Material) serve as points of reference or signposts. For a clinician treating ASD, interpreting the overall importance of Table 4 in Supplementary Material is likely most relevant to the clinician's reference case, in other words, the case the clinician is presently treating.

With the exception of specific developmental delays, almost all psychiatric disorders were diagnosed after ASD ranging from 62 to 98% of the sample for females. Similarly for males, psychiatric disorders followed ASD ranging from 56 to 100% of the sample with index psychiatric diagnoses. Before ASD diagnosis was established, pre-ASD psychiatric morbidity was monotonic, that is only specific developmental delay (315) was diagnosed. Any explanation underpinning these results is speculative. For instance, ASD literacy in the mid-1990s may have been low and not favored in psychiatric curricula or graduate continuing medical education. Given the increase in the annual rate of ASD diagnosis between 1993 and 2010 within the catchment area (28), this explanation is plausible. Furthermore, Child and Adolescent Psychiatry only formed in 2010 as a specialization recognized by the Canadian Royal College of Physicians and Surgeons, which could have influenced the dearth of specialized training and sub-specialization in developmental disorders. Hence, it is likely that psychiatric-training-based recognition of ASD was different in the late 1990's and early 2000's (about 7 years being the average age of ASD diagnosis).

## Limitations

The present work has important limitations. One main limitation of the present study is that each major class or individual ICD diagnosis and specific ICD diagnosis proportions before and after ASD was calculated independently of one another. This limitation was addressed in part by stratifying the main classes by age groups and recording the average age and duration before or after ASD, where ASD and the ICD disorder both occurred in the same individual.

Furthermore, it was not possible to examine the linkage of the dataset employed in this study with databases containing prescription data, laboratory results, or to delve further into treatment outcomes *via* case review, or genetic analysis of biological samples. One contemporary database that links primarily pharmacological prescription data is the United Kingdom's primary care Health Improvement Network (THIN) database (66). A current literature search for the terms “THIN database multimorbidity” produced zero results. A similar search for “THIN database comorbidity” produced 18 results, while “THIN database morbidity” produced 247 results. The majority of these results were focused on a minimal number of associated diseases or disorders and/or prescriptions. Furthermore, evaluation of the THIN database indicated that information loss due to incomplete mapping of medical and drug codes, as well as data structure in the Common Data Model used in THIN limits, its use for all possible epidemiological evaluation studies (66). The database employed in the present study represented the formal physician diagnoses (~90 Million) for the health service seeking population in the Calgary Health Zone (~0.75 Million). As noted in previously published limitations respecting this database, over-diagnosis of common disorders, under-diagnosis of rare disorders and misdiagnosis are all likely represented, albeit minimally, in the data (29, 31). Also, the algorithms applied to the data were developed specifically to examine the temporal associations of the diagnosed diseases and disorders. The approaches taken in analysis with this large dataset are relatively novel, as indicated by the dearth of similar studies, such as those emerging from the THIN database. To date the published studies based on the present database have provided largely broad stroke findings, which, while to some extent useful, are signposts that point to the need for further research and development. The present results extend these findings with a description the temporal order of transient hypermorbidity, not only by the proxy of age for the main ICD classes, but also in relation to the full range of specific ICD diagnoses. The provision of age and duration before and after the pivot ASD add value, possibly bringing information into a framework useful to both research and clinical practice.

The results of population-based analysis of multimorbidity are complicated and not necessarily straight forward or intuitive. The results point to some potential possibilities in terms of mechanism, yet there is no possibility that studies focusing only on associative morbidity are conclusive. As with the studies that illustrate a potential confounding of treatment with outcome diagnoses such as ulcerative colitis or cancer (29, 31), confounding exists between epigenetics, individual differences, diathesis, and, importantly, long-term unstudied adverse effects of treatment. While less likely to be the case with diseases, such as ulcerative colitis, the results of this population-based study, where ASD diagnosis alone is the key variable, must be considered as speculative.

## Conclusions

The present work extends the understanding of temporal, transient hyper-multimorbidity associated with ASD. It confirms the ASD association with gastrointestinal problems and immunological disorders, sensory, and neurological disorders, which have been identified in more constrained studies of comorbidity.

The recently published papers from this population dataset have been informative in terms of signaling the importance of considering mental disorders in relation to physical or biomedical disorders (29–31, 34) and, in the cases of ulcerative colitis and cancer, identifying potential mechanism underlining individual vulnerability (diathesis) or the confounding of the physical or biomedical disease with treatment, or both (29, 31). For example, in both cancers and ulcerative colitis, neuroleptics may disrupt immunological cell-cell communication and represent long-term adverse effects associated with mental disease treatment. This hypothesis is speculative, providing only a direction for detailed basic research.

In conclusion, the present study has the advantage of examining multiform morbidity in a population. The types of analyses including ORs by all ages, stratified by age groups and the proportions before and after any pivot ASD resulted in diagnostic profiles representing novel information potentially informing both basic research, clinical education and practice. The relationship between ASD and the main classes of ICD disorder stratified by age gives a very general indication of the importance of broad diagnostic groupings at different ages and illustrates the need to carefully consider the multiple temporal associations of ASD and specific ICD disorders. The greatest amount of information possibly useful for clinicians is in the proportions of disease before and after ASD, particularly when considered by frequency, age and duration.

## Next steps

It is apparent in considering the study of temporal morbidity in population-based data sets, going forward may require a standardized approach in order for study results to be comparable. Further, noting that the ORs and the specific ICD proportions were independent in the calculation of each main ICD class, hence the resulting ORs (Tables 1–3) and proportions (Table 4 in Supplementary Material) were related only by age and duration before or after ASD. The most important next step in the development of analysis algorithms is to advance an approach to the study of multimorbidity that compares a different matrix format representing the temporal order of specific diagnoses in individuals across all specific ICD diagnoses. The highest levels of observed sequences of disorders that arise and lead to a particular disorder or set of disorders, such as ASD, would represent more a precise analysis than that presently presented. A preliminary algorithm that accomplishes this precision is currently undergoing validation testing.

## Author contributions

DC conceptualized the study, organized the data, conducted analyses, and wrote the paper.

### Conflict of interest statement

The author declares that the research was conducted in the absence of any commercial or financial relationships that could be construed as a potential conflict of interest.

## Footnotes

^1^A deficit of self-awareness, a condition in which a person (in this case system) seems unaware of its existence (e.g., multimorbidity).

^2^This study was approved under Ethics ID: REB_15-1057.
