# Supplementary material for: A 16-Year Cohort Analysis of Autism Spectrum Disorder-Associated Morbidity in a Pediatric Population
Source: Front Psychiatry. 2018 Nov 29;9:635. doi: 10.3389/fpsyt.2018.00635 (PMC6281889; doi:10.3389/fpsyt.2018.00635)
Supplement: Supplementary file 1 [file Table_4.pdf]

Table 4: Comparisons of proportions of ASD arising significantly before and after distinct ICD diagnoses for females and males.

Females: ASD arising significantly before ICD diagnosis.								
Row	ICD Diagnosis	mean age	duration ASD before (days)	ASD arising before (# cases)	duration ASD after (days)	ASD arising after (# cases)	Proportion ASD before ICD diagnosis (LCI, UCI)	Proportion ASD after ICD diagnosis (LCI, UCI)
1	other viral disease (78)	10	-1343	17	865	11	0.61 (0.51, 0.7)	0.39 (0.3, 0.49)
2	benign neoplasm of skin (216)	8	-1255	5	284	2	0.71 (0.54, 0.89)	0.29 (0.11, 0.46)
3	diabetes mellitus (250)	11	-1943	3	0	0	0.75 (0.53, 0.97)	0.25 (0.03, 0.47)
4	psychoses of childhood (299)	9	-879	49	0	0	0.98 (0.96, 1)	0.02 (0, 0.04)
5	neurotic disorders (300)	10	-1155	33	1002	17	0.66 (0.59, 0.73)	0.34 (0.27, 0.41)
6	personality disorders (301)	14	-1597	4	72	1	0.8 (0.62, 0.98)	0.2 (0.02, 0.38)
7	acute reaction to stress (308)	12	-1478	6	306	2	0.75 (0.6, 0.9)	0.25 (0.1, 0.4)
8	adjustment reaction (309)	12	-1275	11	853	6	0.65 (0.53, 0.76)	0.35 (0.24, 0.47)
9	depressive disorder nec (311)	12	-1563	19	462	8	0.7	0.3

							(0.62, 0.79)	(0.21, 0.38)
10	conduct disturbance nec (312)	10	-909	21	872	12	0.64<br>(0.55, 0.72)	0.36<br>(0.28, 0.45)
11	emotional disorder child/adol (313)	9	-1053	34	975	12	0.74<br>(0.67, 0.8)	0.26<br>(0.2, 0.33)
12	hyperkinetic syndrome (314)	9	-1274	37	783	23	0.62<br>(0.55, 0.68)	0.38<br>(0.32, 0.45)
13	mild intellect disability (317)	9	-892	5	1638	1	0.83<br>(0.68, 0.99)	0.17<br>(0.01, 0.32)
14	migraine (346)	9	-942	4	931	1	0.8<br>(0.62, 0.98)	0.2<br>(0.02, 0.38)
15	iris/ciliary body disorder (364)	6	-1115	3	0	0	0.75<br>(0.53, 0.97)	0.25<br>(0.03, 0.47)
16	ill-defined heart disorder (429)	9	-1309	3	0	0	0.75<br>(0.53, 0.97)	0.25<br>(0.03, 0.47)
17	gastritis and duodenitis (535)	9	-687	8	2271	4	0.67<br>(0.53, 0.8)	0.33<br>(0.2, 0.47)
18	noninflamm disorder vagina (623)	13	-1109	3	4147	1	0.75<br>(0.53, 0.97)	0.25<br>(0.03, 0.47)
19	disorder of menstruation (626)	13	-1947	9	1046	3	0.75<br>(0.63, 0.88)	0.25<br>(0.13, 0.38)
20	back disorder nec & nos (724)	8	-1285	5	1775	2	0.71<br>(0.54, 0.89)	0.29<br>(0.11, 0.46)
21	tibia & fibula fracture (823)	10	-1656	5	146	1	0.83<br>(0.68, 0.99)	0.17<br>(0.01, 0.32)
22	sprain wrist & hand (842)	11	-763	5	1364	1	0.83<br>(0.68, 0.99)	0.17<br>(0.01, 0.32)
23	open wound of finger (883)	11	-1183	5	2121	2	0.71<br>(0.54, 0.89)	0.29<br>(0.11, 0.46)
24	injury nec/nos (959)	10	-1526	7	2279	2	0.78<br>(0.64, 0.92)	0.22<br>(0.08, 0.36)
25	vaccin for viral disease (V04)	11	-1679	8	1087	2	0.8<br>(0.67, 0.93)	0.2<br>(0.07, 0.33)

26	administrative encounter (V68)	13	-1033	4	538	1	0.8 (0.62, 0.98)	0.2 (0.02, 0.38)
27	screen-endo/nutr/metab (V77)	10	-1455	3	0	0	0.75 (0.53, 0.97)	0.25 (0.03, 0.47)
28	screen-heart/resp/gu disorder (V81)	10	-1166	10	880	3	0.77 (0.65, 0.89)	0.23 (0.11, 0.35)

Table 4 (Continued) Females: ASD arising significantly after ICD diagnosis.

ICD Diagnosis	mean age	duration ASD before (days)	ASD arising before (# cases)	duration ASD after (days)	ASD arising after (# cases)	Proportion ASD before ICD diagnosis (LCI, UCI)	Proportion ASD after ICD diagnosis (LCI, UCI)
ill-defined intest inf (009)	7	-913	15	1890	43	0.26 (0.2, 0.32)	0.74 (0.68, 0.8)
chickenpox (052)	6	-863	3	2132	11	0.21 (0.1, 0.32)	0.79 (0.68, 0.9)
other viral exanthemata (057)	6	-489	4	1302	8	0.33 (0.2, 0.47)	0.67 (0.53, 0.8)
viral inf in other dis/nos (079)	6	-912	9	1710	22	0.29 (0.21, 0.37)	0.71 (0.63, 0.79)
dermatophytosis (110)	15	-1398	1	2000	9	0.1 (0.01, 0.19)	0.9 (0.81, 0.99)
candidiasis (112)	7	-1190	3	1757	33	0.08 (0.04, 0.13)	0.92 (0.87, 0.96)
fluid/electrolyte disorder (276)	6	-1045	2	1989	15	0.12 (0.04, 0.2)	0.88 (0.8, 0.96)
specific develop delays (315)	8	-1052	20	751	49	0.29 (0.24, 0.34)	0.71 (0.66, 0.76)
infantile cerebral palsy (343)	8	-1037	3	1646	9	0.25 (0.13, 0.38)	0.75 (0.63, 0.88)
corneal opacity/disorder (371)	14	-3595	1	1742	4	0.2	0.8

							(0.02, 0.38)	(0.62, 0.98)
41	disorders of conjunctiva (372)	9	-1656	15	1708	51	0.23<br>(0.18, 0.28)	0.77<br>(0.72, 0.82)
42	disorders of eyelids nec (374)	7	-2273	1	2513	3	0.25<br>(0.03, 0.47)	0.75<br>(0.53, 0.97)
43	lacrimal system disorder (375)	7	-1043	1	1898	6	0.14<br>(0.01, 0.28)	0.86<br>(0.72, 0.99)
44	strabismus (378)	7	-1639	8	1727	13	0.38<br>(0.27, 0.49)	0.62<br>(0.51, 0.73)
45	nonsuppur otitis media (381)	6	-709	12	1553	39	0.24<br>(0.18, 0.29)	0.76<br>(0.71, 0.82)
46	otitis media, suppur/nos (382)	7	-1041	16	1641	73	0.18<br>(0.14, 0.22)	0.82<br>(0.78, 0.86)
47	disorders of ear nec (388)	7	-871	6	1249	13	0.32<br>(0.21, 0.42)	0.68<br>(0.58, 0.79)
48	hearing loss (389)	13	-2035	3	1196	11	0.21<br>(0.1, 0.32)	0.79<br>(0.68, 0.9)
49	acute nasopharyngitis (460)	8	-1105	21	1570	70	0.23<br>(0.19, 0.27)	0.77<br>(0.73, 0.81)
50	acute sinusitis (461)	7	-923	15	1588	25	0.38<br>(0.3, 0.45)	0.63<br>(0.55, 0.7)
51	ac laryngitis/tracheitis (464)	7	-861	14	1647	25	0.36<br>(0.28, 0.44)	0.64<br>(0.56, 0.72)
52	ac up resp inf multiple sites/nos (465)	9	-1216	20	1442	72	0.22<br>(0.17, 0.26)	0.78<br>(0.74, 0.83)
53	ac bronchitis/bronchiol (466)	8	-1162	17	1839	36	0.32<br>(0.26, 0.38)	0.68<br>(0.62, 0.74)
54	chr pharyng/nasopharyng (472)	12	-1155	2	1417	9	0.18<br>(0.07, 0.3)	0.82<br>(0.7, 0.93)
55	other uppr respiratory disorder (478)	10	-1041	2	1706	5	0.29<br>(0.11, 0.46)	0.71<br>(0.54, 0.89)
56	pneumonia, organism nos (486)	11	-1550	6	1603	14	0.3<br>(0.2, 0.4)	0.7<br>(0.6, 0.8)

57	bronchitis nos (490)	10	-1076	5	1306	10	0.33 (0.21, 0.46)	0.67 (0.54, 0.79)
58	asthma (493)	9	-1065	14	1682	30	0.32 (0.25, 0.39)	0.68 (0.61, 0.75)
59	toother develop/erupt disorder (520)	10	-1258	1	2118	12	0.08 (0, 0.15)	0.92 (0.85, 1)
60	hard tissue disorder of teeth (521)	9	-1587	3	1967	7	0.3 (0.16, 0.44)	0.7 (0.56, 0.84)
61	gingival/periodontal disorder (523)	13	-2196	1	2100	3	0.25 (0.03, 0.47)	0.75 (0.53, 0.97)
62	oral soft tissue disease (528)	12	-2351	1	1406	9	0.1 (0.01, 0.19)	0.9 (0.81, 0.99)
63	other noninf gastroenterit (558)	14	-3002	1	2439	19	0.05 (0, 0.1)	0.95 (0.9, 1)
64	other renal & ureteral disorder (593)	4	-729	3	1382	6	0.33 (0.18, 0.49)	0.67 (0.51, 0.82)
65	cystitis (595)	7	-1036	9	1436	22	0.29 (0.21, 0.37)	0.71 (0.63, 0.79)
66	other urinary tract disor (599)	8	-1038	10	1388	23	0.3 (0.22, 0.38)	0.7 (0.62, 0.78)
67	impetigo (684)	12	-2313	2	1715	7	0.22 (0.08, 0.36)	0.78 (0.64, 0.92)
68	erythematosquamous derm (690)	13	-2478	1	1884	7	0.13 (0.01, 0.24)	0.88 (0.76, 0.99)
69	atopic dermatitis (691)	11	-1145	7	1546	26	0.21 (0.14, 0.28)	0.79 (0.72, 0.86)
70	contact dermatitis (692)	11	-1644	13	1235	45	0.22 (0.17, 0.28)	0.78 (0.72, 0.83)
71	other dermatoses (702)	14	-2761	1	821	4	0.2 (0.02, 0.38)	0.8 (0.62, 0.98)
72	other cervical spine disorder (723)	10	-1474	2	1681	5	0.29 (0.11, 0.46)	0.71 (0.54, 0.89)
73	disorder of muscle/lig/fascia (728)	9	-1704	1	1069	5	0.17 (0.01, 0.32)	0.83 (0.68, 0.99)

74	cong anom ear/face/neck (744)	11	-1727	1	2016	3	0.25 (0.03, 0.47)	0.75 (0.53, 0.97)
75	general symptoms (780)	9	-1198	31	1471	60	0.34 (0.29, 0.39)	0.66 (0.61, 0.71)
76	nerv/musculskel sys symp (781)	10	-1077	7	1149	29	0.19 (0.13, 0.26)	0.81 (0.74, 0.87)
77	nutrit/metab/devel symp (783)	9	-938	8	1659	36	0.18 (0.12, 0.24)	0.82 (0.76, 0.88)
78	cardiovascular sys symp (785)	9	-1100	2	1640	18	0.1 (0.03, 0.17)	0.9 (0.83, 0.97)
79	resp sys/other chest symp (786)	8	-1054	16	1931	33	0.33 (0.26, 0.39)	0.67 (0.61, 0.74)
80	gi system symptoms (787)	9	-1124	16	1761	30	0.35 (0.28, 0.42)	0.65 (0.58, 0.72)
81	humerus fracture (812)	10	-610	1	1691	3	0.25 (0.03, 0.47)	0.75 (0.53, 0.97)
82	radius & ulna fracture (813)	12	-1801	2	1261	5	0.29 (0.11, 0.46)	0.71 (0.54, 0.89)
83	elbow dislocation (832)	3	-405	1	1865	3	0.25 (0.03, 0.47)	0.75 (0.53, 0.97)
84	sprain of back nec/nos (847)	12	-1662	4	1212	10	0.29 (0.16, 0.41)	0.71 (0.59, 0.84)
85	other open wound of head (873)	5	-467	7	1138	23	0.23 (0.16, 0.31)	0.77 (0.69, 0.84)
86	superficial injury head (910)	2	-241	1	2187	5	0.17 (0.01, 0.32)	0.83 (0.68, 0.99)
87	superficial injury trunk (911)	12	-2238	1	1775	4	0.2 (0.02, 0.38)	0.8 (0.62, 0.98)
88	superficial inj of hand (914)	3	-314	2	1494	5	0.29 (0.11, 0.46)	0.71 (0.54, 0.89)
89	contusion face/scalp/nck (920)	10	-1529	2	1182	9	0.18 (0.07, 0.3)	0.82 (0.7, 0.93)
90	contusion of upper limb (923)	15	-1709	2	863	6	0.25 (0.1, 0.4)	0.75 (0.6, 0.9)

91	certain adverse eff nec (995)	9	-1569	10	1803	19	0.34 (0.26, 0.43)	0.66 (0.57, 0.74)
92	other hx of health hazards (V15)	12	-1324	4	1177	11	0.27 (0.15, 0.38)	0.73 (0.62, 0.85)
93	health supervision child (V20)	8	-1148	18	2018	82	0.18 (0.14, 0.22)	0.82 (0.78, 0.86)
94	encountr proc/aftrcr nec (V58)	10	-835	3	1275	6	0.33 (0.18, 0.49)	0.67 (0.51, 0.82)
95	follow-up examination (V67)	9	-325	2	1526	6	0.25 (0.1, 0.4)	0.75 (0.6, 0.9)
96	general medical exam (V70)	10	-1709	24	1628	36	0.4 (0.34, 0.46)	0.6 (0.54, 0.66)
97	observation-suspect cond (V71)	9	-443	2	1706	6	0.25 (0.1, 0.4)	0.75 (0.6, 0.9)
98	screening-mental disorder (V79)	8	-797	1	1014	5	0.17 (0.01, 0.32)	0.83 (0.68, 0.99)
99	whooping cough (033)	0	0	0	3187	4	0.2 (0.02, 0.38)	0.8 (0.62, 0.98)
100	other intest helminthiases (127)	2	0	0	1602	3	0.25 (0.03, 0.47)	0.75 (0.53, 0.97)
101	pediculosis and phthirus (132)	3	0	0	2497	6	0.14 (0.01, 0.28)	0.86 (0.72, 0.99)
102	hemangioma/lymphangioma (228)	1	0	0	2272	3	0.25 (0.03, 0.47)	0.75 (0.53, 0.97)
103	disorders of the globe (360)	5	0	0	1113	3	0.25 (0.03, 0.47)	0.75 (0.53, 0.97)
104	keratitis (370)	4	0	0	1926	4	0.2 (0.02, 0.38)	0.8 (0.62, 0.98)
105	deviated nasal septum (470)	7	0	0	1276	4	0.2 (0.02, 0.38)	0.8 (0.62, 0.98)
106	bronchopneumonia org nos (485)	1	0	0	1728	3	0.25 (0.03, 0.47)	0.75 (0.53, 0.97)
107	other resp system diseases (519)	1	0	0	2529	4	0.2 (0.02, 0.38)	0.8 (0.62, 0.98)

108	stomach function disorder (536)	2	0	0	2707	3	0.25 (0.03, 0.47)	0.75 (0.53, 0.97)
109	other intestinal disorders (569)	1	0	0	1146	3	0.25 (0.03, 0.47)	0.75 (0.53, 0.97)
110	erythematous conditions (695)	3	0	0	1223	7	0.13 (0.01, 0.24)	0.88 (0.76, 0.99)
111	cardiac septal clos anom (745)	1	0	0	2018	3	0.25 (0.03, 0.47)	0.75 (0.53, 0.97)
112	cong musculoskel deform (754)	1	0	0	1755	11	0.08 (0, 0.16)	0.92 (0.84, 1)
113	disorders relating to short gestation and unspecified low birthweight (764)	0	0	0	3182	3	0.25 (0.03, 0.47)	0.75 (0.53, 0.97)
114	disorders relating to short gestation and unspecified low birthweight (765)	0	0	0	2308	6	0.14 (0.01, 0.28)	0.86 (0.72, 0.99)
115	other nb respiratory cond (770)	0	0	0	2247	10	0.09 (0, 0.18)	0.91 (0.82, 1)
116	perinatal infection (771)	0	0	0	1267	3	0.25 (0.03, 0.47)	0.75 (0.53, 0.97)
117	other perinatal jaundice (774)	0	0	0	2018	9	0.1 (0.01, 0.19)	0.9 (0.81, 0.99)
118	nb endocrin/metabol disorder (775)	0	0	0	1226	4	0.2 (0.02, 0.38)	0.8 (0.62, 0.98)
119	other perinatal condition (779)	0	0	0	2259	14	0.07 (0, 0.13)	0.93 (0.87, 1)
120	abn find-body struct nos (793)	3	0	0	1503	3	0.25 (0.03, 0.47)	0.75 (0.53, 0.97)
121	sprain elbow & forearm (841)	7	0	0	584	3	0.25 (0.03, 0.47)	0.75 (0.53, 0.97)
122	superficial inj foot/toe (917)	4	0	0	1796	3	0.25 (0.03, 0.47)	0.75 (0.53, 0.97)
123	normal pregnancy (V22)	1	0	0	659	3	0.25 (0.03, 0.47)	0.75 (0.53, 0.97)
124	single liveborn (V30)	0	0	0	2212	52	0.02 (0, 0.04)	0.98 (0.96, 1)

125	other psychosocial circum (V62))	5	0	0	783	3	0.25 (0.03, 0.47)	0.75 (0.53, 0.97)

Table 4 (Continued) Males: ASD arising significantly before ICD diagnosis.

ICD Diagnosis	mean age	duration ASD before (days)	ASD arising before (# cases)	duration ASD after (days)	ASD arising after (# cases)	Proportion ASD before ICD diagnosis (LCI, UCI)	Proportion ASD after ICD diagnosis (LCI, UCI)
other viral disease (78)	9	-1280	94	1208	74	0.56 (0.52, 0.6)	0.44 (0.4, 0.48)
benign neoplasm of skin (216)	11	-1411	27	1036	16	0.63 (0.55, 0.7)	0.37 (0.3, 0.45)
obesity/hyperaliment (278)	12	-2054	17	2369	3	0.85 (0.77, 0.93)	0.15 (0.07, 0.23)
affective psychoses (296)	12	-754	7	585	1	0.88 (0.76, 0.99)	0.13 (0.01, 0.24)
other nonorganic psychoses (298)	14	-1768	5	623	1	0.83 (0.68, 0.99)	0.17 (0.01, 0.32)
psychoses of childhood (299)	8	-793	288	0	0	1 (0.99, 1)	0 (0, 0.01)
special symptom nec (307)	10	-1112	45	1201	36	0.56 (0.5, 0.61)	0.44 (0.39, 0.5)
depressive disorder nec (311)	11	-1099	74	853	45	0.62 (0.58, 0.67)	0.38 (0.33, 0.42)
hyperkinetic syndrome (314)	10	-1085	221	782	137	0.62 (0.59, 0.64)	0.38 (0.36, 0.41)
mild intellect disabilty (317)	9	-1151	14	834	6	0.7	0.3

							(0.6, 0.8)	(0.2, 0.4)
138	migraine (346)	11	-1293	12	1012	6	0.67<br>(0.56, 0.78)	0.33<br>(0.22, 0.44)
139	essential hypertension (401)	10	-1330	5	2695	1	0.83<br>(0.68, 0.99)	0.17<br>(0.01, 0.32)
140	disease of capillaries (448)	10	-1099	3	205	1	0.75<br>(0.53, 0.97)	0.25<br>(0.03, 0.47)
141	gastric ulcer (531)	6	-726	3	0	0	0.75<br>(0.53, 0.97)	0.25<br>(0.03, 0.47)
142	other bladder disorders (596)	12	-1572	4	305	1	0.8<br>(0.62, 0.98)	0.2<br>(0.02, 0.38)
143	other skin hypertro/atroph (701)	10	-1621	13	1533	8	0.62<br>(0.51, 0.73)	0.38<br>(0.27, 0.49)
144	diseases of nail (703)	11	-1673	19	1852	9	0.68<br>(0.59, 0.77)	0.32<br>(0.23, 0.41)
145	sebaceous gland disease (706)	12	-1861	40	2491	19	0.68<br>(0.62, 0.74)	0.32<br>(0.26, 0.38)
146	diff connective tiss disorder (710)	9	-1816	3	837	1	0.75<br>(0.53, 0.97)	0.25<br>(0.03, 0.47)
147	internal derangemnt knee (717)	14	-1706	5	1384	2	0.71<br>(0.54, 0.89)	0.29<br>(0.11, 0.46)
148	joint disorder nec & nos (719)	10	-1250	28	1389	20	0.58<br>(0.51, 0.65)	0.42<br>(0.35, 0.49)
149	periph enthesopathies (726)	11	-2018	3	457	1	0.75<br>(0.53, 0.97)	0.25<br>(0.03, 0.47)
150	other soft tissue disorder (729)	10	-1220	66	1345	51	0.56<br>(0.52, 0.61)	0.44<br>(0.39, 0.48)
151	osteomyelitis (730)	9	-770	3	0	0	0.75<br>(0.53, 0.97)	0.25<br>(0.03, 0.47)
152	osteochondropathies (732)	13	-1554	4	1133	1	0.8<br>(0.62, 0.98)	0.2<br>(0.02, 0.38)
153	flat foot (734)	11	-1439	12	1231	6	0.67<br>(0.56, 0.78)	0.33<br>(0.22, 0.44)

154	curvature of spine (737)	15	-2601	5	1685	2	0.71 (0.54, 0.89)	0.29 (0.11, 0.46)
155	radius & ulna fracture (813)	10	-1420	30	1176	21	0.59 (0.52, 0.66)	0.41 (0.34, 0.48)
156	metacarpal fracture (815)	13	-2133	6	1175	2	0.75 (0.6, 0.9)	0.25 (0.1, 0.4)
157	ankle fracture (824)	11	-884	5	1523	2	0.71 (0.54, 0.89)	0.29 (0.11, 0.46)
158	sprain wrist & hand (842)	11	-1765	24	1151	10	0.71 (0.63, 0.78)	0.29 (0.22, 0.37)
159	sprain of hip & thigh (843)	11	-2047	6	888	2	0.75 (0.6, 0.9)	0.25 (0.1, 0.4)
160	sprain of ankle & foot (845)	11	-1259	32	925	21	0.6 (0.54, 0.67)	0.4 (0.33, 0.46)
161	sprain nec (848)	11	-1203	8	1676	4	0.67 (0.53, 0.8)	0.33 (0.2, 0.47)
162	vaccin for viral disease (V04)	9	-1182	27	1118	19	0.59 (0.51, 0.66)	0.41 (0.34, 0.49)
163	other psychosocial circum (V62)	5	-715	4	0	0	0.8 (0.62, 0.98)	0.2 (0.02, 0.38)
164	administrative encounter (V68)	11	-1068	18	622	9	0.67 (0.58, 0.76)	0.33 (0.24, 0.42)
165	screen-heart/resp/gu disorder (V81)	10	-1088	55	769	31	0.64 (0.59, 0.69)	0.36 (0.31, 0.41)

Table 4: (Continued) Males ASD arising significantly after ICD diagnosis.

ICD Diagnosis	mean age	duration ASD before (days)	ASD arising before (# cases)	duration ASD after (days)	ASD arising after (# cases)	Proportion ASD before ICD diagnosis (LCI, UCI)	Proportion ASD after ICD diagnosis (LCI, UCI)
intestinal infection nec (008)	1	-308	1	1735	9	0.1 (0.01, 0.19)	0.9 (0.81, 0.99)
ill-defined intest inf (009)	7	-709	58	1885	335	0.15 (0.13, 0.17)	0.85 (0.83, 0.87)
whooping cough (33)	5	-547	2	1786	8	0.2 (0.07, 0.33)	0.8 (0.67, 0.93)
strep throat/scarlet fev (34)	8	-937	24	1395	45	0.35 (0.29, 0.41)	0.65 (0.59, 0.71)
septicemia (38)	7	-805	3	1959	15	0.17 (0.08, 0.25)	0.83 (0.75, 0.92)
chickenpox (52)	7	-717	20	1731	75	0.21 (0.17, 0.25)	0.79 (0.75, 0.83)
herpes zoster (53)	12	-836	6	1286	14	0.3 (0.2, 0.4)	0.7 (0.6, 0.8)
herpes simplex (54)	8	-1104	6	1408	19	0.24 (0.15, 0.33)	0.76 (0.67, 0.85)
rubella (56)	7	-1270	1	1545	6	0.14 (0.01, 0.28)	0.86 (0.72, 0.99)
other viral exanthemata (57)	7	-539	20	1599	61	0.25	0.75

							(0.2, 0.29)	(0.71, 0.8)
178	coxsackie viral disease (74)	8	-820	4	1784	17	0.19<br>(0.1, 0.28)	0.81<br>(0.72, 0.9)
179	viral inf in other dis/nos (79)	8	-842	38	1858	133	0.22<br>(0.19, 0.25)	0.78<br>(0.75, 0.81)
180	dermatophytosis (110)	9	-1703	8	1902	32	0.2<br>(0.14, 0.26)	0.8<br>(0.74, 0.86)
181	candidiasis (112)	7	-744	8	2228	158	0.05<br>(0.03, 0.06)	0.95<br>(0.94, 0.97)
182	pediculosis and phthirus (132)	7	-571	5	1833	15	0.25<br>(0.15, 0.35)	0.75<br>(0.65, 0.85)
183	diabetes mellitus (250)	13	-2611	3	1615	6	0.33<br>(0.18, 0.49)	0.67<br>(0.51, 0.82)
184	other endocrine disorders (259)	9	-1292	1	1215	5	0.17<br>(0.01, 0.32)	0.83<br>(0.68, 0.99)
185	fluid/electrolyte disorder (276)	7	-1135	14	1465	54	0.21<br>(0.16, 0.25)	0.79<br>(0.75, 0.84)
186	metabolism disorder nec/nos (277)	4	-97	2	2148	7	0.22<br>(0.08, 0.36)	0.78<br>(0.64, 0.92)
187	iron deficiency anemias (280)	11	-1434	4	1165	22	0.15<br>(0.08, 0.22)	0.85<br>(0.78, 0.92)
188	white blood cell disorders (288)	6	-1286	1	2297	4	0.2<br>(0.02, 0.38)	0.8<br>(0.62, 0.98)
189	other blood disease (289)	8	-1271	4	1326	15	0.21<br>(0.12, 0.3)	0.79<br>(0.7, 0.88)
190	acute reaction to stress (308)	10	-887	9	1409	18	0.33<br>(0.24, 0.42)	0.67<br>(0.58, 0.76)
191	specific develop delays (315)	9	-915	130	855	243	0.35<br>(0.32, 0.37)	0.65<br>(0.63, 0.68)
192	meningitis, unspecified (322)	11	-1209	1	1163	3	0.25<br>(0.03, 0.47)	0.75<br>(0.53, 0.97)
193	epilepsy (345)	9	-1555	20	1691	28	0.42<br>(0.35, 0.49)	0.58<br>(0.51, 0.65)

194	other brain conditions (348)	4	-98	2	1663	9	0.18 (0.07, 0.3)	0.82 (0.7, 0.93)
195	retinal detachment (361)	6	-1008	1	1190	3	0.25 (0.03, 0.47)	0.75 (0.53, 0.97)
196	retinal disorders nec (362)	10	-1414	11	1802	33	0.25 (0.18, 0.32)	0.75 (0.68, 0.82)
197	iris/ciliary body disorder (364)	6	-713	1	1315	4	0.2 (0.02, 0.38)	0.8 (0.62, 0.98)
198	blindness and low vision (369)	6	-1135	1	1630	4	0.2 (0.02, 0.38)	0.8 (0.62, 0.98)
199	keratitis (370)	7	-941	2	1114	5	0.29 (0.11, 0.46)	0.71 (0.54, 0.89)
200	corneal opacity/disorder (371)	9	-1381	8	1571	19	0.3 (0.21, 0.38)	0.7 (0.62, 0.79)
201	disorders of conjunctiva (372)	8	-1005	94	1671	270	0.26 (0.24, 0.28)	0.74 (0.72, 0.76)
202	inflammation of eyelids (373)	9	-1311	21	1695	34	0.38 (0.32, 0.45)	0.62 (0.55, 0.68)
203	lacrimal system disorder (375)	2	-157	1	1975	25	0.04 (0, 0.08)	0.96 (0.92, 1)
204	strabismus (378)	9	-1051	55	1350	83	0.4 (0.36, 0.44)	0.6 (0.56, 0.64)
205	eye disorders nec (379)	8	-834	7	1642	15	0.32 (0.22, 0.42)	0.68 (0.58, 0.78)
206	disorder of external ear (380)	8	-1075	66	1437	123	0.35 (0.31, 0.38)	0.65 (0.62, 0.69)
207	nonsuppur otitis media (381)	7	-791	78	1768	230	0.25 (0.23, 0.28)	0.75 (0.72, 0.77)
208	otitis media, suppur/nos (382)	6	-678	92	1765	401	0.19 (0.17, 0.2)	0.81 (0.8, 0.83)
209	disorders of ear nec (388)	8	-1089	29	1535	58	0.33 (0.28, 0.38)	0.67 (0.62, 0.72)
210	hearing loss (389)	8	-740	7	1602	40	0.15 (0.1, 0.2)	0.85 (0.8, 0.9)

211	other endocardial disease (424)	5	-39	1	2110	4	0.2 (0.02, 0.38)	0.8 (0.62, 0.98)
212	intracranial hem nec/nos (432)	11	-1211	1	1079	3	0.25 (0.03, 0.47)	0.75 (0.53, 0.97)
213	acute nasopharyngitis (460)	8	-1027	78	1777	386	0.17 (0.15, 0.19)	0.83 (0.81, 0.85)
214	acute sinusitis (461)	9	-1137	48	1462	113	0.3 (0.26, 0.33)	0.7 (0.67, 0.74)
215	acute pharyngitis (462)	9	-1113	135	1538	222	0.38 (0.35, 0.4)	0.62 (0.6, 0.65)
216	acute tonsillitis (463)	8	-922	69	1489	149	0.32 (0.29, 0.35)	0.68 (0.65, 0.71)
217	ac laryngitis/tracheitis (464)	7	-1042	42	1661	158	0.21 (0.18, 0.24)	0.79 (0.76, 0.82)
218	ac up resp inf multiple sites/nos (465)	7	-959	104	1825	397	0.21 (0.19, 0.23)	0.79 (0.77, 0.81)
219	ac bronchitis/bronchiol (466)	8	-1031	84	1813	263	0.24 (0.22, 0.27)	0.76 (0.73, 0.78)
220	deviated nasal septum (470)	8	-1360	2	1316	8	0.2 (0.07, 0.33)	0.8 (0.67, 0.93)
221	chr pharyng/nasopharyng (472)	7	-810	15	1654	32	0.32 (0.25, 0.39)	0.68 (0.61, 0.75)
222	chronic sinusitis (473)	8	-1115	17	1296	32	0.35 (0.28, 0.41)	0.65 (0.59, 0.72)
223	chr t & a disorder (474)	8	-851	34	1338	56	0.38 (0.33, 0.43)	0.62 (0.57, 0.67)
224	other uppr respiratory disorder (478)	10	-1684	7	1920	20	0.26 (0.17, 0.34)	0.74 (0.66, 0.83)
225	viral pneumonia (480)	4	-135	2	1006	10	0.17 (0.06, 0.27)	0.83 (0.73, 0.94)
226	other bacterial pneumonia (482)	10	-675	2	1470	19	0.1 (0.03, 0.16)	0.9 (0.84, 0.97)
227	bronchopneumonia org nos (485)	9	-943	5	2529	11	0.31 (0.2, 0.43)	0.69 (0.57, 0.8)

228	pneumonia, organism nos (486)	8	-1037	26	1690	72	0.27 (0.22, 0.31)	0.73 (0.69, 0.78)
229	influenza (487)	9	-1136	41	1721	67	0.38 (0.33, 0.43)	0.62 (0.57, 0.67)
230	bronchitis nos (490)	8	-1183	18	1713	64	0.22 (0.17, 0.27)	0.78 (0.73, 0.83)
231	chronic bronchitis (491)	15	-1890	1	2400	19	0.05 (0, 0.1)	0.95 (0.9, 1)
232	asthma (493)	8	-1090	87	1674	214	0.29 (0.26, 0.32)	0.71 (0.68, 0.74)
233	other resp system diseases (519)	7	-1249	9	1706	19	0.32 (0.23, 0.41)	0.68 (0.59, 0.77)
234	toother develop/erupt disorder (520)	6	-869	6	2477	59	0.09 (0.06, 0.13)	0.91 (0.87, 0.94)
235	gingival/periodontal disorder (523)	8	-1308	4	1908	11	0.27 (0.15, 0.38)	0.73 (0.62, 0.85)
236	other dental disorder (525)	12	-1145	2	1454	8	0.2 (0.07, 0.33)	0.8 (0.67, 0.93)
237	oral soft tissue disease (528)	10	-1264	16	1801	34	0.32 (0.25, 0.39)	0.68 (0.61, 0.75)
238	tongue disorders (529)	14	-1021	1	1947	18	0.05 (0, 0.1)	0.95 (0.9, 1)
239	diseases of esophagus (530)	8	-1109	3	1911	26	0.1 (0.05, 0.16)	0.9 (0.84, 0.95)
240	gastritis and duodenitis (535)	8	-1157	11	1895	23	0.32 (0.24, 0.4)	0.68 (0.6, 0.76)
241	stomach function disorder (536)	11	-1997	3	1874	7	0.3 (0.16, 0.44)	0.7 (0.56, 0.84)
242	other gastroduodenal disorder (537)	6	-213	1	3212	5	0.17 (0.01, 0.32)	0.83 (0.68, 0.99)
243	acute appendicitis (540)	7	-1328	3	850	6	0.33 (0.18, 0.49)	0.67 (0.51, 0.82)
244	inguinal hernia (550)	6	-969	2	2442	34	0.06 (0.02, 0.09)	0.94 (0.91, 0.98)

245	other abdominal hernia (553)	5	-411	2	1840	14	0.13 (0.04, 0.21)	0.88 (0.79, 0.96)
246	other noninf gastroenterit (558)	6	-1010	11	1956	107	0.09 (0.07, 0.12)	0.91 (0.88, 0.93)
247	intestinal obstruction (560)	9	-2298	2	1643	6	0.25 (0.1, 0.4)	0.75 (0.6, 0.9)
248	function digestive disorder nec (564)	8	-974	50	1969	112	0.31 (0.27, 0.34)	0.69 (0.66, 0.73)
249	anal fissure & fistula (565)	9	-1298	2	2022	14	0.13 (0.04, 0.21)	0.88 (0.79, 0.96)
250	other intestinal disorders (569)	12	-2548	3	2663	10	0.23 (0.11, 0.35)	0.77 (0.65, 0.89)
251	gastrointestinal hemorrhage (578)	10	-926	2	1550	7	0.22 (0.08, 0.36)	0.78 (0.64, 0.92)
252	intestinal malabsorption (579)	6	-541	6	1912	17	0.26 (0.17, 0.35)	0.74 (0.65, 0.83)
253	kidney infection (590)	9	-1241	3	1192	8	0.27 (0.14, 0.41)	0.73 (0.59, 0.86)
254	other renal & ureteral disorder (593)	8	-1082	1	1347	21	0.05 (0, 0.09)	0.95 (0.91, 1)
255	cystitis (595)	8	-1154	18	1426	40	0.31 (0.25, 0.37)	0.69 (0.63, 0.75)
256	urethral stricture (598)	5	-182	1	2186	3	0.25 (0.03, 0.47)	0.75 (0.53, 0.97)
257	other urinary tract disor (599)	8	-887	26	1555	60	0.3 (0.25, 0.35)	0.7 (0.65, 0.75)
258	hydrocele (603)	8	-1011	6	2077	43	0.12 (0.08, 0.17)	0.88 (0.83, 0.92)
259	redundant prepuce & phimosis (605)	8	-761	12	1865	45	0.21 (0.16, 0.26)	0.79 (0.74, 0.84)
260	disorders of penis (607)	8	-1198	14	1869	69	0.17 (0.13, 0.21)	0.83 (0.79, 0.87)
261	other disorders of male genital organs (608)	9	-1098	10	1737	19	0.34 (0.26, 0.43)	0.66 (0.57, 0.74)

262	other breast disorders (611)	13	-2397	2	1012	5	0.29 (0.11, 0.46)	0.71 (0.54, 0.89)
263	carbuncle and furuncle (680)	11	-428	4	1813	9	0.31 (0.18, 0.44)	0.69 (0.56, 0.82)
264	other cellulitis/abscess (682)	9	-1243	54	1596	90	0.38 (0.33, 0.42)	0.63 (0.58, 0.67)
265	acute lymphadenitis (683)	9	-857	3	2282	6	0.33 (0.18, 0.49)	0.67 (0.51, 0.82)
266	impetigo (684)	8	-1212	25	1322	45	0.36 (0.3, 0.41)	0.64 (0.59, 0.7)
267	other local skin infection (686)	9	-1067	16	1579	47	0.25 (0.2, 0.31)	0.75 (0.69, 0.8)
268	erythematosquamous derm (690)	10	-703	5	2276	15	0.25 (0.15, 0.35)	0.75 (0.65, 0.85)
269	atopic dermatitis (691)	8	-1130	41	1926	111	0.27 (0.23, 0.31)	0.73 (0.69, 0.77)
270	contact dermatitis (692)	8	-1024	78	1901	206	0.27 (0.25, 0.3)	0.73 (0.7, 0.75)
271	dermat d/t intern agent (693)	12	-1600	4	1385	12	0.25 (0.14, 0.36)	0.75 (0.64, 0.86)
272	erythematous conditions (695)	9	-659	4	1608	15	0.21 (0.12, 0.3)	0.79 (0.7, 0.88)
273	pruritus & like cond (698)	8	-1518	2	1438	8	0.2 (0.07, 0.33)	0.8 (0.67, 0.93)
274	corns and callosities (700)	6	-414	1	1082	6	0.14 (0.01, 0.28)	0.86 (0.72, 0.99)
275	other dermatoses (702)	12	-1927	7	1652	15	0.32 (0.22, 0.42)	0.68 (0.58, 0.78)
276	disorders of sweat gland (705)	14	-3861	1	2246	4	0.2 (0.02, 0.38)	0.8 (0.62, 0.98)
277	urticaria (708)	8	-887	28	1653	44	0.39 (0.33, 0.45)	0.61 (0.55, 0.67)
278	other skin disorders (709)	9	-1251	33	1729	70	0.32 (0.27, 0.37)	0.68 (0.63, 0.73)

279	other cervical spine disorder (723)	8	-832	14	1146	25	0.36 (0.28, 0.44)	0.64 (0.56, 0.72)
280	back disorder nec & nos (724)	10	-970	15	1884	21	0.42 (0.33, 0.5)	0.58 (0.5, 0.67)
281	other acquired deformity (738)	13	-1241	1	3261	5	0.17 (0.01, 0.32)	0.83 (0.68, 0.99)
282	somatic dysfunction (739)	9	-1144	29	1211	46	0.39 (0.33, 0.44)	0.61 (0.56, 0.67)
283	other nervous system anom (742)	9	-1586	3	1504	9	0.25 (0.13, 0.38)	0.75 (0.63, 0.88)
284	cardiac septal clos anom (745)	14	-1877	1	2097	22	0.04 (0, 0.09)	0.96 (0.91, 1)
285	other cong circ syst anom (747)	4	-83	1	2140	18	0.05 (0, 0.1)	0.95 (0.9, 1)
286	genital organ anomalies (752)	7	-932	8	1946	19	0.3 (0.21, 0.38)	0.7 (0.62, 0.79)
287	urinary system anomalies (753)	10	-47	1	2327	4	0.2 (0.02, 0.38)	0.8 (0.62, 0.98)
288	cong musculoskel deform (754)	9	-1209	5	2217	34	0.13 (0.07, 0.18)	0.87 (0.82, 0.93)
289	other congen limb anomaly (755)	10	-2783	1	1508	6	0.14 (0.01, 0.28)	0.86 (0.72, 0.99)
290	congen skin anomalies (757)	5	-172	1	1386	3	0.25 (0.03, 0.47)	0.75 (0.53, 0.97)
291	chromosomal anomalies (758)	10	-642	3	1301	7	0.3 (0.16, 0.44)	0.7 (0.56, 0.84)
292	congen anomalies nec/nos (759)	10	-1343	2	1872	10	0.17 (0.06, 0.27)	0.83 (0.73, 0.94)
293	disorders relating to short gestation and unspecified low birthweight (765)	11	-160	1	2249	60	0.02 (0, 0.03)	0.98 (0.97, 1)
294	general symptoms (780)	9	-1179	123	1637	354	0.26 (0.24, 0.28)	0.74 (0.72, 0.76)
295	nerv/musculskel sys symp (781)	10	-1369	40	1649	95	0.3 (0.26, 0.34)	0.7 (0.66, 0.74)

296	skin/other integument symp (782)	9	-1129	73	1792	216	0.25 (0.23, 0.28)	0.75 (0.72, 0.77)
297	nutrit/metab/devel symp (783)	8	-1009	20	1962	173	0.1 (0.08, 0.13)	0.9 (0.87, 0.92)
298	symptoms invol head/neck (784)	9	-1018	76	1365	154	0.33 (0.3, 0.36)	0.67 (0.64, 0.7)
299	cardiovascular sys symp (785)	9	-1227	23	1812	96	0.19 (0.16, 0.23)	0.81 (0.77, 0.84)
300	resp sys/other chest symp (786)	10	-1318	89	1703	211	0.3 (0.27, 0.32)	0.7 (0.68, 0.73)
301	gi system symptoms (787)	8	-1217	41	1956	152	0.21 (0.18, 0.24)	0.79 (0.76, 0.82)
302	urinary system symptoms (788)	8	-997	38	1786	64	0.37 (0.32, 0.42)	0.63 (0.58, 0.68)
303	other abdomen/pelvis symp (789)	9	-1389	89	1560	145	0.38 (0.35, 0.41)	0.62 (0.59, 0.65)
304	abnormal blood findings (790)	9	-1090	9	1926	24	0.27 (0.2, 0.35)	0.73 (0.65, 0.8)
305	skull vault fracture (800)	12	-1919	1	1454	3	0.25 (0.03, 0.47)	0.75 (0.53, 0.97)
306	tibia & fibula fracture (823)	9	-797	5	1888	13	0.28 (0.17, 0.38)	0.72 (0.62, 0.83)
307	fx of tarsal/metatarsal (825)	10	-1512	3	947	6	0.33 (0.18, 0.49)	0.67 (0.51, 0.82)
308	concussion (850)	9	-1158	12	1580	22	0.35 (0.27, 0.43)	0.65 (0.57, 0.73)
309	other brain injury (854)	9	-1037	13	1706	57	0.19 (0.14, 0.23)	0.81 (0.77, 0.86)
310	other open wound of head (873)	8	-1115	56	1628	134	0.29 (0.26, 0.33)	0.71 (0.67, 0.74)
311	open wound site nec (879)	8	-693	2	1721	6	0.25 (0.1, 0.4)	0.75 (0.6, 0.9)
312	open wound of finger (883)	9	-1012	21	1396	29	0.42 (0.35, 0.49)	0.58 (0.51, 0.65)

313	traum amputation finger (886)	13	-1555	1	2018	3	0.25 (0.03, 0.47)	0.75 (0.53, 0.97)
314	open wound of toe (893)	6	-350	4	1739	8	0.33 (0.2, 0.47)	0.67 (0.53, 0.8)
315	superficial injury head (910)	7	-1052	22	1707	62	0.26 (0.21, 0.31)	0.74 (0.69, 0.79)
316	superficial inj finger (915)	7	-936	5	2044	15	0.25 (0.15, 0.35)	0.75 (0.65, 0.85)
317	superficial inj foot/toe (917)	3	-279	1	1181	8	0.11 (0.01, 0.22)	0.89 (0.78, 0.99)
318	superfic inj eye/adnexa (918)	8	-582	5	1588	10	0.33 (0.21, 0.46)	0.67 (0.54, 0.79)
319	superficial inj other site (919)	7	-982	7	1941	16	0.3 (0.21, 0.4)	0.7 (0.6, 0.79)
320	contusion face/scalp/nck (920)	8	-832	16	1907	62	0.21 (0.16, 0.25)	0.79 (0.75, 0.84)
321	contusion of upper limb (923)	11	-1882	15	1070	25	0.38 (0.3, 0.45)	0.63 (0.55, 0.7)
322	crushing inj upper limb (927)	9	-1348	2	1613	5	0.29 (0.11, 0.46)	0.71 (0.54, 0.89)
323	foreign body extern eye (930)	8	-1531	3	1206	8	0.27 (0.14, 0.41)	0.73 (0.59, 0.86)
324	foreign body in nose (932)	6	-819	5	1538	18	0.22 (0.13, 0.3)	0.78 (0.7, 0.87)
325	burn of head/face/neck (941)	13	-1553	2	1433	5	0.29 (0.11, 0.46)	0.71 (0.54, 0.89)
326	burn of arm (943)	8	-854	2	1222	7	0.22 (0.08, 0.36)	0.78 (0.64, 0.92)
327	burn of hand & wrist (944)	12	-1149	6	1916	21	0.22 (0.14, 0.3)	0.78 (0.7, 0.86)
328	pois-analgesic/antipyret (965)	5	-772	1	2643	4	0.2 (0.02, 0.38)	0.8 (0.62, 0.98)
329	pois-medicinal nec/nos (977)	6	-585	5	1549	20	0.2 (0.12, 0.28)	0.8 (0.72, 0.88)

330	certain adverse eff nec (995)	8	-918	45	1665	100	0.31 (0.27, 0.35)	0.69 (0.65, 0.73)
331	surg compl-body syst nec (997)	12	-2893	1	1141	4	0.2 (0.02, 0.38)	0.8 (0.62, 0.98)
332	other surgical compl nec (998)	8	-1268	5	1849	15	0.25 (0.15, 0.35)	0.75 (0.65, 0.85)
333	other vaccin for singl disorder (V05)	8	-564	2	866	7	0.22 (0.08, 0.36)	0.78 (0.64, 0.92)
334	prophylactic measures (V07)	10	-981	11	1196	18	0.38 (0.29, 0.47)	0.62 (0.53, 0.71)
335	other hx of health hazards (V15)	7	-1016	16	1359	28	0.36 (0.29, 0.44)	0.64 (0.56, 0.71)
336	health supervision child (V20)	9	-1075	51	2050	481	0.1 (0.08, 0.11)	0.9 (0.89, 0.92)
337	constitut state in devel (V21)	8	-953	1	1584	15	0.06 (0, 0.12)	0.94 (0.88, 1)
338	normal pregnancy (V22)	6	-538	1	2090	11	0.08 (0, 0.16)	0.92 (0.84, 1)
339	procreative management (V26)	7	-563	1	1605	4	0.2 (0.02, 0.38)	0.8 (0.62, 0.98)
340	prob w special functions (V41)	7	-1544	1	1844	3	0.25 (0.03, 0.47)	0.75 (0.53, 0.97)
341	head/neck/trunk problems (V48)	14	-3783	1	1771	5	0.17 (0.01, 0.32)	0.83 (0.68, 0.99)
342	atten to artificial open (V55)	8	-599	2	752	7	0.22 (0.08, 0.36)	0.78 (0.64, 0.92)
343	encountr proc/aftrcr nec (V58)	9	-831	14	1421	37	0.27 (0.21, 0.34)	0.73 (0.66, 0.79)
344	other family circumstances (V61)	6	-747	4	1401	13	0.24 (0.13, 0.34)	0.76 (0.66, 0.87)
345	other reason for consult (V65)	10	-1161	65	1655	119	0.35 (0.32, 0.39)	0.65 (0.61, 0.68)
346	follow-up examination (V67)	10	-1192	13	1654	39	0.25 (0.19, 0.31)	0.75 (0.69, 0.81)

347	general medical exam (V70)	9	-1389	99	1486	203	0.33 (0.3, 0.35)	0.67 (0.65, 0.7)
348	special examinations (V72)	8	-885	36	1360	59	0.38 (0.33, 0.43)	0.62 (0.57, 0.67)
349	screening-mental disorder (V79)	6	-590	5	907	33	0.13 (0.08, 0.19)	0.87 (0.81, 0.92)
350	screen-neuro/eye/ear disorder (V80)	6	-513	1	1345	6	0.14 (0.01, 0.28)	0.86 (0.72, 0.99)
351	other intest helminthiases (127)	3	0	0	1437	3	0.25 (0.03, 0.47)	0.75 (0.53, 0.97)
352	lipoma (214)	9	0	0	539	3	0.25 (0.03, 0.47)	0.75 (0.53, 0.97)
353	other ben neo soft tissue (215)	7	0	0	529	4	0.2 (0.02, 0.38)	0.8 (0.62, 0.98)
354	other pancreatic disorder (251)	2	0	0	2012	6	0.14 (0.01, 0.28)	0.86 (0.72, 0.99)
355	other deficiency anemia (281)	3	0	0	1836	4	0.2 (0.02, 0.38)	0.8 (0.62, 0.98)
356	purpura & other hemor cond (287)	3	0	0	1411	11	0.08 (0, 0.16)	0.92 (0.84, 1)
357	psychic factor w other disorder (316)	8	0	0	947	3	0.25 (0.03, 0.47)	0.75 (0.53, 0.97)
358	bacterial meningitis (320)	1	0	0	3106	3	0.25 (0.03, 0.47)	0.75 (0.53, 0.97)
359	muscular dystrophies (359)	0	0	0	1904	3	0.25 (0.03, 0.47)	0.75 (0.53, 0.97)
360	cataract (366)	2	0	0	1462	4	0.2 (0.02, 0.38)	0.8 (0.62, 0.98)
361	mastoiditis et al (383)	5	0	0	2035	10	0.09 (0, 0.18)	0.91 (0.82, 1)
362	hypertensive renal disorder (403)	5	0	0	1789	3	0.25 (0.03, 0.47)	0.75 (0.53, 0.97)
363	chr laryng/laryngotrach (476)	3	0	0	1806	3	0.25 (0.03, 0.47)	0.75 (0.53, 0.97)

364	pneumococcal pneumonia (481)	4	0	0	1137	4	0.2 (0.02, 0.38)	0.8 (0.62, 0.98)
365	pneum in other infec disorder (484)	3	0	0	2015	3	0.25 (0.03, 0.47)	0.75 (0.53, 0.97)
366	chr airway obstruct nec (496)	2	0	0	1759	9	0.1 (0.01, 0.19)	0.9 (0.81, 0.99)
367	other lung diseases (518)	0	0	0	1677	14	0.07 (0, 0.13)	0.93 (0.87, 1)
368	regional enteritis (555)	0	0	0	1986	3	0.25 (0.03, 0.47)	0.75 (0.53, 0.97)
369	peritonitis (567)	3	0	0	1882	4	0.2 (0.02, 0.38)	0.8 (0.62, 0.98)
370	hydronephrosis (591)	2	0	0	2189	3	0.25 (0.03, 0.47)	0.75 (0.53, 0.97)
371	urethritis/urethral synd (597)	6	0	0	1603	4	0.2 (0.02, 0.38)	0.8 (0.62, 0.98)
372	other female genital disorder (629)	3	0	0	1218	3	0.25 (0.03, 0.47)	0.75 (0.53, 0.97)
373	other congen heart anom (746)	3	0	0	1937	6	0.14 (0.01, 0.28)	0.86 (0.72, 0.99)
374	respiratory syst anomaly (748)	1	0	0	2137	14	0.07 (0, 0.13)	0.93 (0.87, 1)
375	other upper gi anomaly (750)	1	0	0	2435	13	0.07 (0, 0.14)	0.93 (0.86, 1)
376	other anom digestive syst (751)	0	0	0	1434	3	0.25 (0.03, 0.47)	0.75 (0.53, 0.97)
377	other musculoskelet anomal (756)	0	0	0	1912	10	0.09 (0, 0.18)	0.91 (0.82, 1)
378	maternal compl aff nb (761)	0	0	0	2547	4	0.2 (0.02, 0.38)	0.8 (0.62, 0.98)
379	other compl deliv aff nb (763)	0	0	0	2433	12	0.08 (0, 0.15)	0.92 (0.85, 1)
380	disorders relating to short gestation and unspecified low birthweight (764)	0	0	0	2406	12	0.08 (0, 0.15)	0.92 (0.85, 1)

381	long gestat/high birthwt (766)	0	0	0	2863	5	0.17 (0.01, 0.32)	0.83 (0.68, 0.99)
382	intrauterine asphyxia (768)	0	0	0	2664	7	0.13 (0.01, 0.24)	0.88 (0.76, 0.99)
383	respiratory distress syn (769)	0	0	0	2362	19	0.05 (0, 0.1)	0.95 (0.9, 1)
384	other nb respiratory cond (770)	0	0	0	2336	46	0.02 (0, 0.04)	0.98 (0.96, 1)
385	perinatal infection (771)	0	0	0	3256	13	0.07 (0, 0.14)	0.93 (0.86, 1)
386	nb hemolyt dis:isoimmu (773)	0	0	0	1949	5	0.17 (0.01, 0.32)	0.83 (0.68, 0.99)
387	other perinatal jaundice (774)	0	0	0	2263	70	0.01 (0, 0.03)	0.99 (0.97, 1)
388	nb endocrin/metabol disorder (775)	0	0	0	2139	12	0.08 (0, 0.15)	0.92 (0.85, 1)
389	hematological disorder of nb (776)	0	0	0	2187	4	0.2 (0.02, 0.38)	0.8 (0.62, 0.98)
390	perinatal gi system disorder (777)	0	0	0	2351	10	0.09 (0, 0.18)	0.91 (0.82, 1)
391	other perinatal condition (779)	0	0	0	2385	92	0.01 (0, 0.02)	0.99 (0.98, 1)
392	elbow dislocation (832)	2	0	0	1638	12	0.08 (0, 0.15)	0.92 (0.85, 1)
393	sprain sacroiliac region (846)	3	0	0	1890	6	0.14 (0.01, 0.28)	0.86 (0.72, 0.99)
394	late eff musculoskel inj (905)	4	0	0	1621	4	0.2 (0.02, 0.38)	0.8 (0.62, 0.98)
395	burn of trunk (942)	3	0	0	1375	6	0.14 (0.01, 0.28)	0.86 (0.72, 0.99)
396	burn of leg (945)	3	0	0	2107	7	0.13 (0.01, 0.24)	0.88 (0.76, 0.99)
397	alcohol toxicity (980)	3	0	0	1790	3	0.25 (0.03, 0.47)	0.75 (0.53, 0.97)

398	complic medical care nec (999)	1	0	0	2569	4	0.2 (0.02, 0.38)	0.8 (0.62, 0.98)
399	infectious disorder carrier (V02)	4	0	0	1399	3	0.25 (0.03, 0.47)	0.75 (0.53, 0.97)
400	single liveborn (V30)	0	0	0	2450	295	0 (0, 0.01)	1 (0.99, 1)
401	twin, mate liveborn (V31)	0	0	0	2965	3	0.25 (0.03, 0.47)	0.75 (0.53, 0.97)
